# Supplementary material for: Targeting Phosphopeptide Recognition by the Human BRCA1 Tandem BRCT Domain to Interrupt BRCA1-Dependent Signaling
Source: Cell Chem Biol. 2018 Jun 21;25(6):677–690.e12. doi: 10.1016/j.chembiol.2018.02.012 (PMC6015222; doi:10.1016/j.chembiol.2018.02.012)
Supplement: Document S1. Figures S1–S4 [file mmc1.pdf]

Cell Chemical Biology, Volume *25*

Supplemental Information

Targeting Phosphopeptide Recognition by the Human BRCA1 Tandem BRCT Domain to Interrupt BRCA1-Dependent Signaling

Jayaprakash Periasamy, Vadiraj Kurdekar, Subbarao Jasti, Mamatha B. Nijaguna, Sanjana Boggaram, Manjunath A. Hurakadli, Dhruv Raina, Lokavya Meenakshi Kurup, Chetan Chintha, Kavyashree Manjunath, Aneesh Goyal, Gayathri Sadasivam, Kavitha Bharatham, Muralidhara Padigaru, Vijay Potluri, and Ashok R. Venkitaraman

SUPPLEMENTAL INFORMATION

Figure S1. Related to Figure 1. Structure-activity relationships of compounds inhibiting phosphopeptide recognition by the BRCA1 tBRCT. The FP assay used for compound library screening was validated by: (A) Testing the ability of an unlabeled BACH1 phosphopeptide (GGSRST(pSer)PTFNK) to compete for binding with an identical but TAMRA-labeled phosphopeptide to BRCA1 tBRCT. (B) Testing the ability of previously reported oligopeptide inhibitors (Yuan et al., 2011) to inhibit the binding of TAMRA-labeled BACH1 phosphopeptide to BRCA1 tBRCT. In both A and B, percent inhibition is plotted against peptide concentration and IC_{50} was calculated. Experiments shown represent the mean of three independent experiments ± SD. (C) Determination of the Z Factor. The dot plot shows FP values for the binding of TAMRA-labeled BACH1 phosphopeptide to BRCA1 tBRCT (9360 wells from 260 assay plates; mean FP bound = 151±18 mP) compared to similar values after the addition of excess unlabelled phosphopeptide (2600 wells from 260 assay plates; mean FP unbound = 41±7mP). Each dot represents the mean from a single plate. The assay Z Factor was calculated to be 0.65 using the equation Z Factor = 1 − (3xSD bound + 3xSD unbound)/(mP bound − mP unbound), where SD is the standard deviation from the mean, and mP is the mean fluorescence polarization. (D) The Alpha screen assay was validated by testing the ability of previously reported oligopeptide inhibitors (Yuan et al., 2011) to inhibit the binding of biotin-labeled BACH1 phosphopeptide to BRCA1 tBRCT. Percent inhibition is plotted against peptide concentration. Experiments shown represent mean of three independent experiments ± SD. (E) Superimposition of the unliganded form (PDB: 1JNX, cyan) and the peptide-bound form (PDB: 3K0K, grey) of BRCA1 tBRCT confirms the absence of conformational changes after substrate engagement. Key residues in the binding pocket are shown as sticks. (F) Two-dimensional representation of the percentage intermolecular interaction between CCBT002 and residues in the BRCA1 tBRCT binding pocket during a 5 ns MD simulation. Hydrogen bond

interactions are shown in magenta, and the hydrophobic and charged pocket residues are shown in green and purple spheres, respectively. The histogram shows the fraction of protein–ligand contacts between CCBT002 and BRCA1 tBRCT through a 5 ns MD simulation with the pocket residues plotted on X-axis, and the interaction fraction plotted on Y-axis. (G) A dose response curve for the interaction between BACH1 phosphopeptide and BRCA1 tBRCT (labeled with NT-647-NHS fluorescent dye) determined by MST. Peptide concentration is plotted on the X-axis against changes in normalized fluorescence (ΔF_{norm}) on the Y-axis. Experiments shown represent mean of three independent experiments ±SD. (H) Dose response curve for the competitive inhibition of the interaction between BACH1 phosphopeptide and BRCA1 tBRCT by the oligopeptide inhibitor, Ac-pSPVF-COOH, measured by MST. Peptide concentration is plotted on the X-axis against changes in normalized fluorescence (ΔF_{norm}) on the Y-axis. Experiments shown represent mean of three independent experiments ±SD.

Figure S1

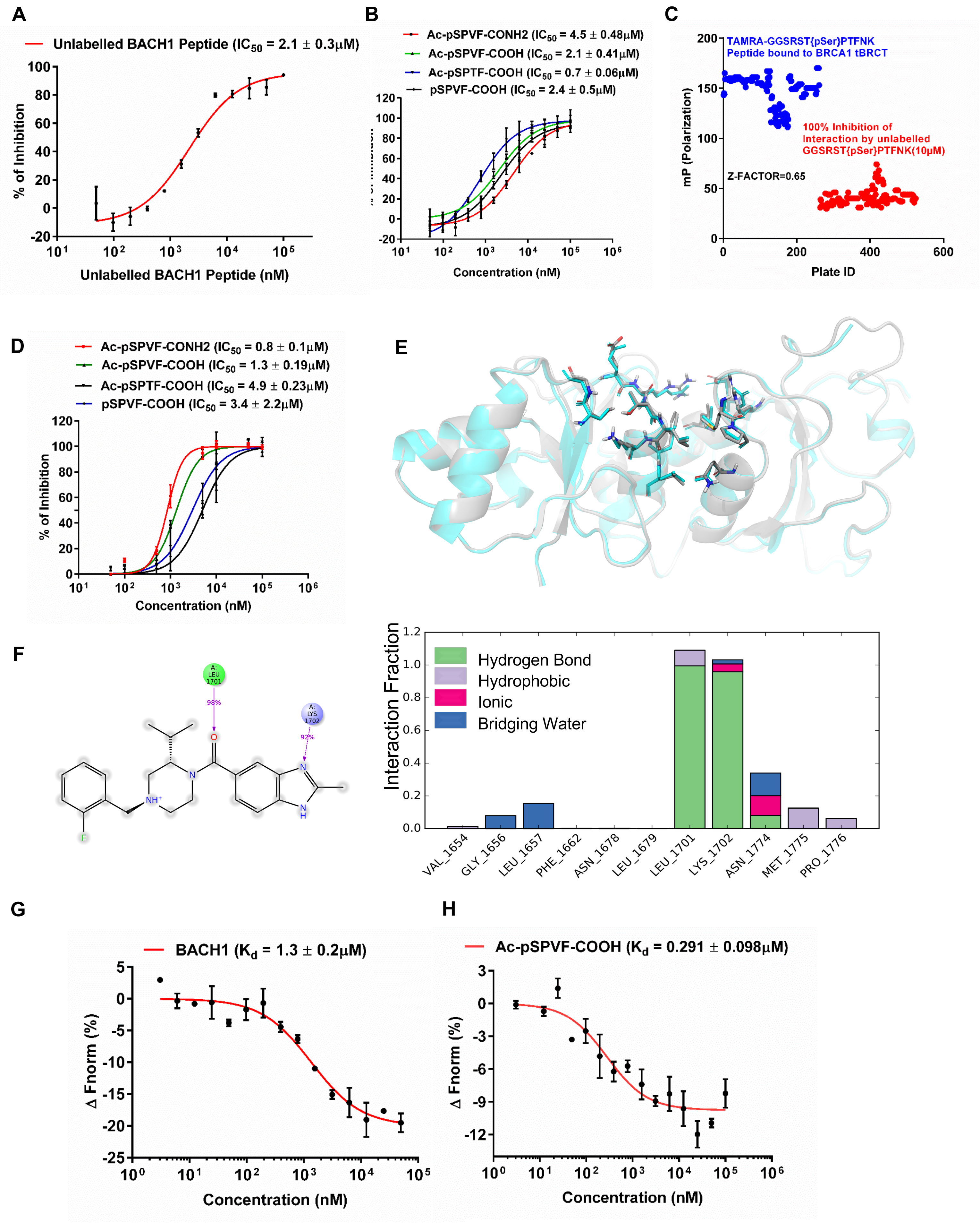

A
Unlabelled BACH1 Peptide (IC50 = 2.1 ± 0.3μM)
% of Inhibition
Unlabelled BACH1 Peptide (nM)
B
Ac-pSPVF-CONH2 (IC50 = 4.5 ± 0.48μM)
Ac-pSPVF-COOH (IC50 = 2.1 ± 0.41μM)
Ac-pSPTF-COOH (IC50 = 0.7 ± 0.06μM)
pSPVF-COOH (IC50 = 2.4 ± 0.5μM)
Concentration (nM)
C
TAMRA-GGSRST{pSer}PTFNK
Peptide bound to BRCA1 tBRCT
100% Inhibition of
Interaction by unlabelled
GGSRST{pSer}PTFNK(10μM)
Z-FACTOR=0.65
mP (Polarization)
Plate ID
D
Ac-pSPVF-CONH2 (IC50 = 0.8 ± 0.1μM)
Ac-pSPVF-COOH (IC50 = 1.3 ± 0.19μM)
Ac-pSPTF-COOH (IC50 = 4.9 ± 0.23μM)
pSPVF-COOH (IC50 = 3.4 ± 2.2μM)
% of Inhibition
Concentration (nM)
E
F
A: LEU 1701
98%
A: LYS 1702
92%
Hydrogen Bond
Hydrophobic
Ionic
Bridging Water
Interaction Fraction
VAL_1654
GLY_1656
LEU_1657
PHE_1662
ASN_1678
LEU_1679
LEU_1701
LYS_1702
ASN_1774
MET_1775
PRO_1776
G
BACH1 (Kd = 1.3 ± 0.2μM)
Δ Fnorm (%)
Concentration (nM)
H
Ac-pSPVF-COOH (Kd = 0.291 ± 0.098μM)
Δ Fnorm (%)
Concentration (nM)

Figure S2, related to Figure 4. Bractoppin inhibits substrate recognition by the BRCA1 tBRCT in the cellular milieu. Effect of Bractoppin (30μM or 100μM) or its inactive analog CCBT2047 (100μM) on FRET measured by sensitized emission. The panel on the left indicates changes in FRET efficiency (mean ± SEM) in cells after 24 h of compound treatment. The second panel on the right, measures FRET efficiency as a dot plot wherein each dot represents a single cell (Control, n= 747; Bractoppin 30μM, 566; Bractoppin 100μM, 789; CCBT2047 100μM, 517). Statistical significance was determined using an unpaired two-tailed t-test *** $p \leq 0.001$.

Figure S2

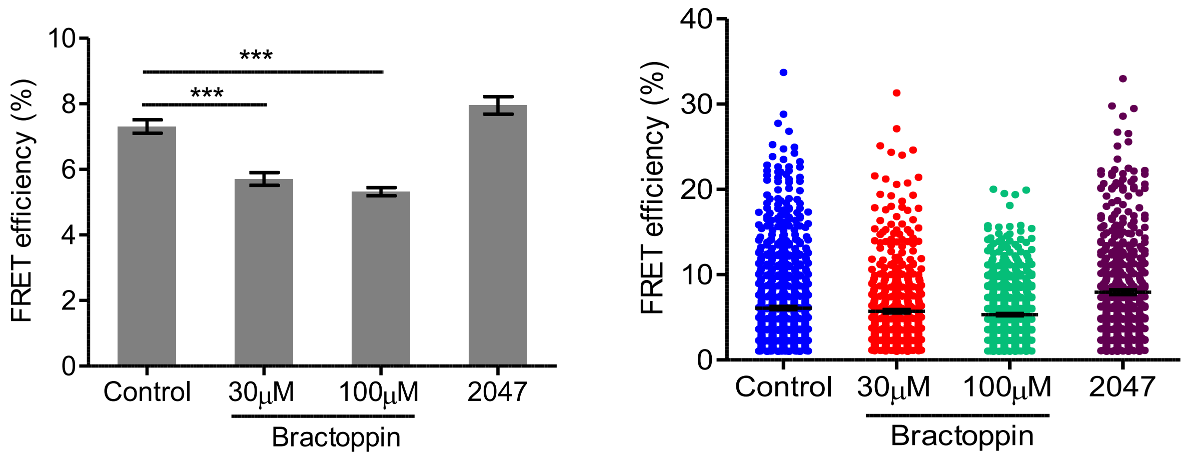

FRET efficiency (%)
10
8
6
4
2
0
***
***
Control
30μM
100μM
2047
Bractoppin
FRET efficiency (%)
40
30
20
10
0
Control
30μM
100μM
2047
Bractoppin

Figure S3, related to Figure 5. Bractoppin selectively inhibits cellular substrate recognition by the tBRCT domain family. Effect of mutations in the BRCA1 tBRCT domains on substrate recognition and the assembly of endogenous BRCA1 foci measured by high-content automated imaging 18 h after the indicated treatments (untreated cells (0 Gy); irradiation alone (16 Gy); Tet-induced expression of wild-type or mutant forms of BRCA1 tBRCT 30h before irradiation). The percentage of cells positive for radiation-induced nuclear BRCA1 foci is shown after expression of (A) wild-type BRCA1 tBRCT (B) mutant BRCA1 tBRCT (M1775R) or (C) mutant BRCA1 tBRCT (S1655A/K1702M). Values are expressed as mean±SD (n= 20000, 0Gy; 15000, 16Gy; 10000, WT BRCA1 tBRCT; 15000, M1775R BRCA1 tBRCT; 15000, S1655A/K1702M BRCA1 tBRCT, Statistical significance *** P≤ 0.001). (D) Percentage of CAL-51 clone60 cells positive for radiation-induced nuclear BRCA1 foci (mean±SD; n= 1472, 0Gy; 1789, 16Gy; 2233, Bractoppin;) enumerated by high-content imaging at low-magnification (see Methods). Treatment conditions were as described in Figure 5A. Statistical significance was determined using an unpaired two-tailed t-test. * p=0.05 (E) Representative high-content microscopic images depicting recruitment of MDC1 and BRCA1 proteins into nuclear foci in the indicated treatment conditions (untreated cells (0Gy); irradiation alone (16Gy); Tet-induced MDC1-tBRCT expression 24h before irradiation; 100µM Bractoppin or its inactive analog CCBT2047 added 6h after irradiation). Staining was done 18h after irradiation in the 1st to 3rd column (left to right) for DAPI (blue), MDC1 (green), BRCA1 (red) and merged images, respectively. Scale bar, 50µm. (F) Percentage of cells positive for radiation-induced TOPBP1 foci without or after treatment with 100µM Bractoppin or its inactive analog CCBT2047 added 6h after irradiation in U2OS cells. Results were measured and depicted as above. Statistical significance was tested using an unpaired two-tailed t-test. ** p≤0.01. Similar results were observed in 3 independent repeats.

Figure S3

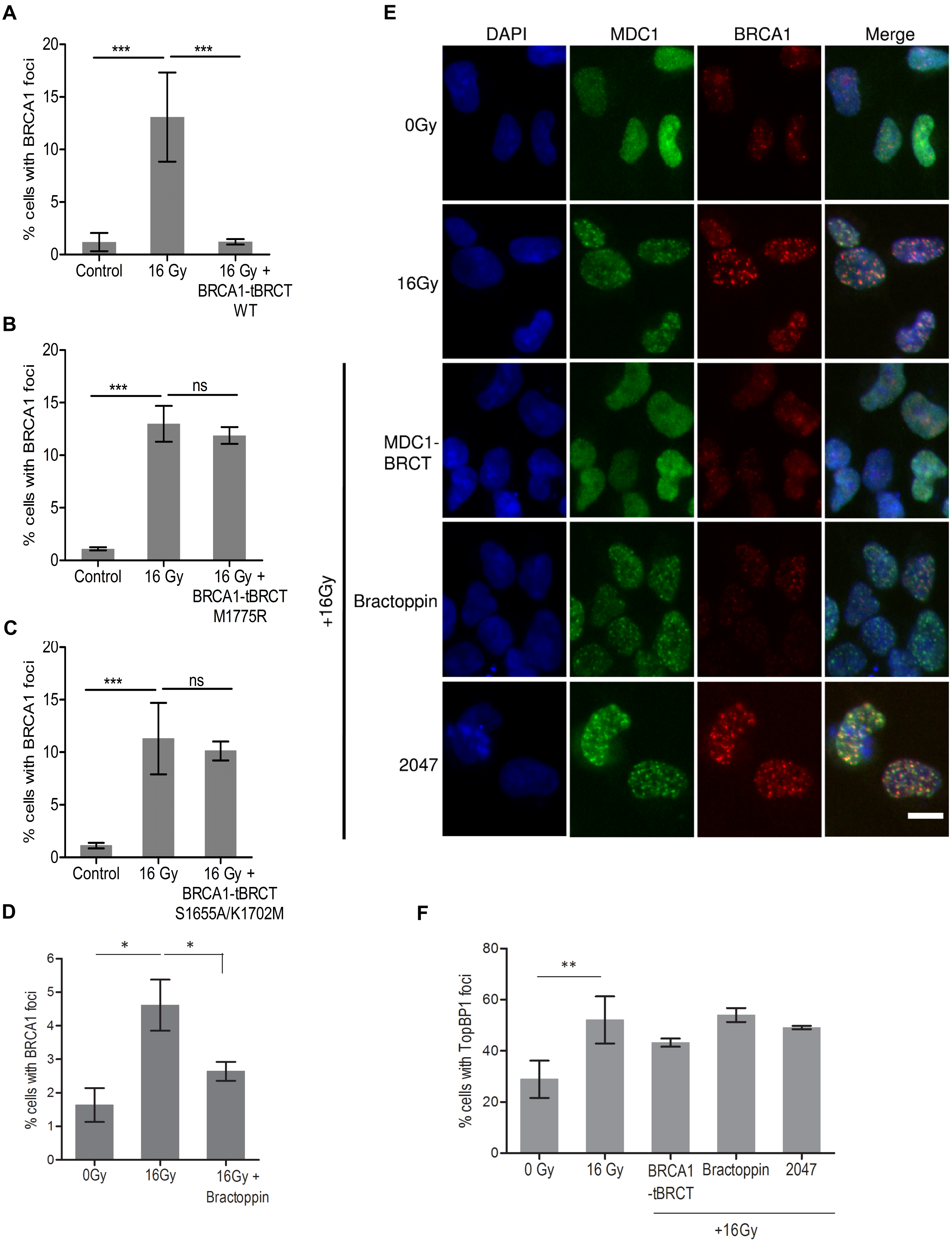
A
% cells with BRCA1 foci
***
***
Control
16 Gy
16 Gy + BRCA1-tBRCT WT
B
% cells with BRCA1 foci
***
ns
Control
16 Gy
16 Gy + BRCA1-tBRCT M1775R
C
% cells with BRCA1 foci
***
ns
Control
16 Gy
16 Gy + BRCA1-tBRCT S1655A/K1702M
D
% cells with BRCA1 foci
*
*
0Gy
16Gy
16Gy + Bractoppin
E
DAPI
MDC1
BRCA1
Merge
0Gy
16Gy
MDC1-BRCT
Bractoppin
2047
+16Gy
F
% cells with TopBP1 foci
**
0 Gy
16 Gy
BRCA1 -tBRCT
Bractoppin
2047
+16Gy

Figure S4. Related to Figure 6. Bractoppin interrupts DNA damage signaling for G2 arrest. Effect of mutations in the BRCA1 tBRCT domains on G2 checkpoint enforcement were measured by flow cytometry after DAPI staining. Cells were irradiated with 4Gy at 8h after synchronous release into the cell cycle from thymidine block, and measurements made 16h later. Wild-type or mutant forms of BRCA1 tBRCT were induced 32h before irradiation. Approximately 15K cells were analyzed per condition, in replicates of 3. (A, B) Percentage of cells (mean ±SD) in the G2/M phase after the indicated treatments. Control, un-irradiated cells; BRCA1 tBRCT S1655A/K1702M double mutant (DM); BRCA1 tBRCT M1775R single mutant (SM). Controls are compared with ± BRCA1 tBRCT DM or SM in the presence of IR. Cells are induced for expression 32h prior to 4Gy IR and measurements are made 16 h post IR. Data quantitatively measures change in accumulation of 4N population (G2+M) across different treatment groups following DAPI staining for its nuclear content. Approximately 15K cells are analyzed per condition, in replicates of 3 using flow cytometer and analyzed using Dean-Jet algorithm for quantitative measurements (mean±SD). Statistical significance was determined using Dunnett's multiple comparisons test post one-way ANOVA. *** P-value: ≤0.001.

(C,D) Cell cycle histograms representing G1 (green), S (yellow) or G2/M (blue) phases after the indicated treatments. Results are representative of 3 independent experiments.

Figure S4

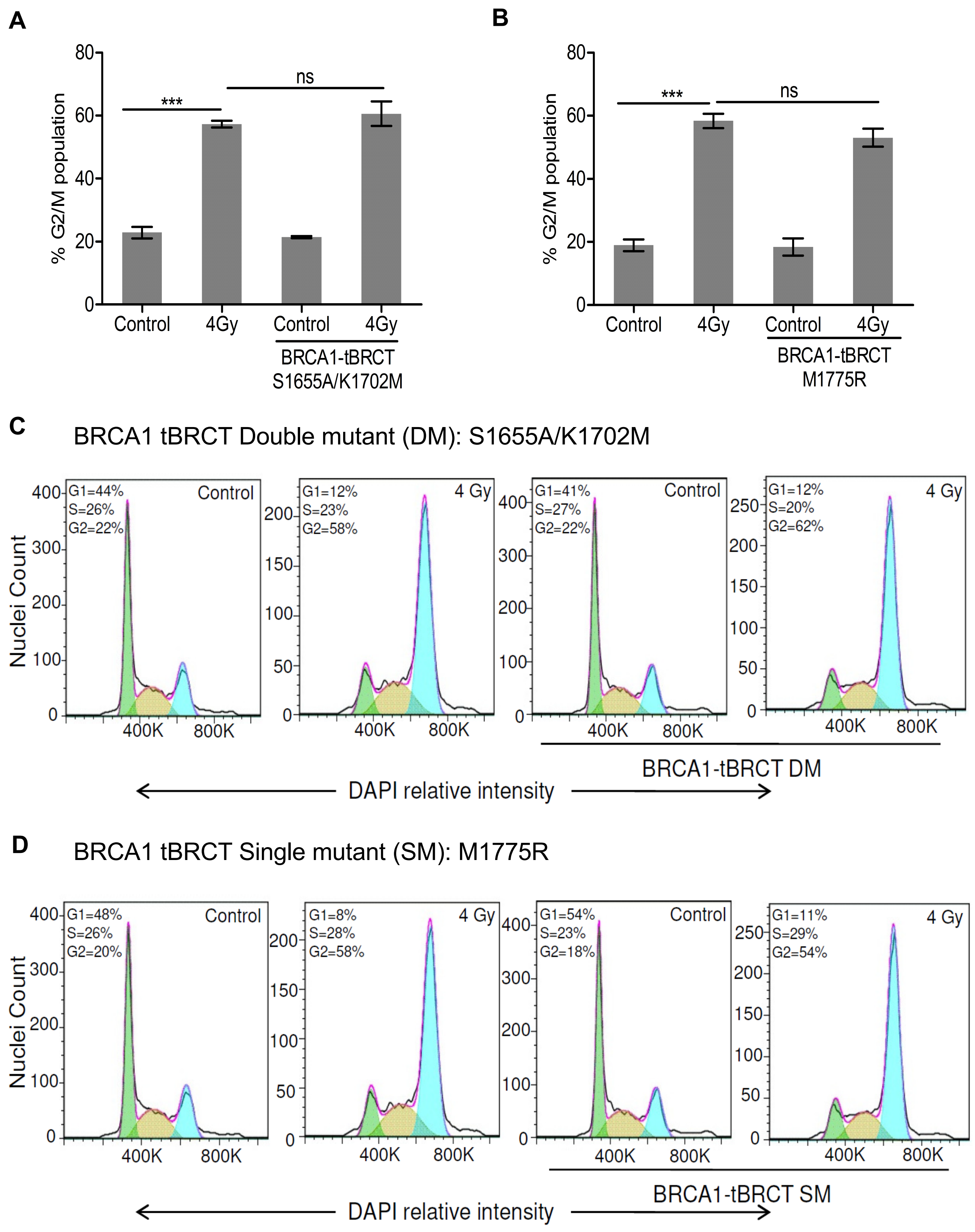
A
B
% G2/M population
***
ns
Control
4Gy
BRCA1-tBRCT
S1655A/K1702M
M1775R
C
BRCA1 tBRCT Double mutant (DM): S1655A/K1702M
Nuclei Count
G1=44%
S=26%
G2=22%
G1=12%
S=23%
G2=58%
G1=41%
S=27%
G2=22%
G1=12%
S=20%
G2=62%
4 Gy
400K
800K
BRCA1-tBRCT DM
DAPI relative intensity
D
BRCA1 tBRCT Single mutant (SM): M1775R
G1=48%
S=26%
G2=20%
G1=8%
S=28%
G2=58%
G1=54%
S=23%
G2=18%
G1=11%
S=29%
G2=54%
BRCA1-tBRCT SM
