# Supplementary material for: Targeting Phosphopeptide Recognition by the Human BRCA1 Tandem BRCT Domain to Interrupt BRCA1-Dependent Signaling
Source: Cell Chem Biol. 2018 Jun 21;25(6):677–690.e12. doi: 10.1016/j.chembiol.2018.02.012 (PMC6015222; doi:10.1016/j.chembiol.2018.02.012)
Supplement: Document S2. Article plus Supplemental Information [file mmc2.pdf]

Article

Cell Chemical Biology

Targeting Phosphopeptide Recognition by the Human BRCA1 Tandem BRCT Domain to Interrupt BRCA1-Dependent Signaling

Graphical Abstract

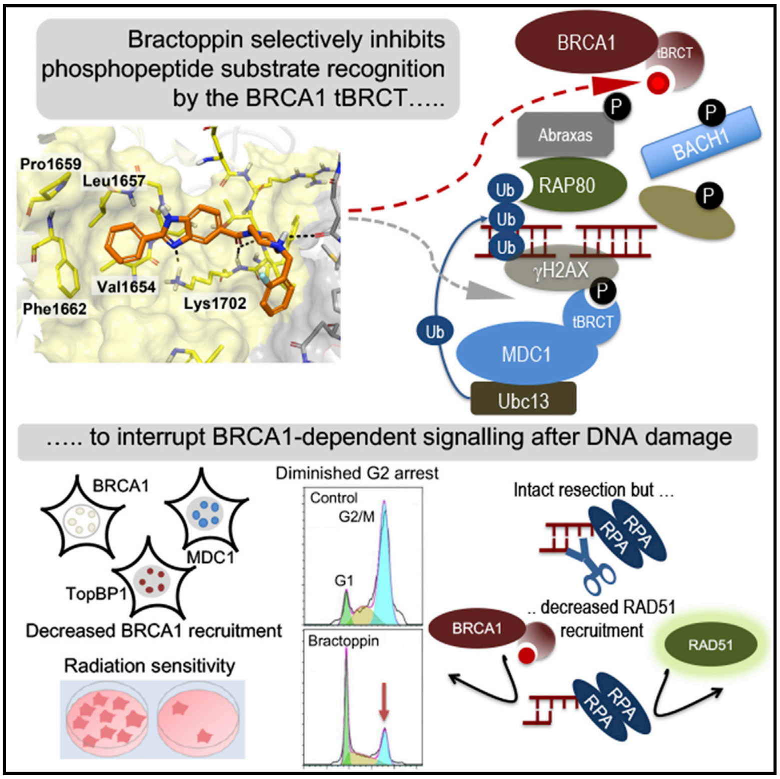

Highlights

- Bractoppin selectively blocks phosphopeptide recognition by the BRCA1 tBRCT domain
- Bractoppin engages tBRCT residues recognizing pSer, plus an adjacent pocket
- Bractoppin interrupts BRCA1 tBRCT-dependent cellular signals evoked by DNA damage
- This work opens avenues to inhibit intracellular signaling by the tBRCT domain family

Authors

Jayaprakash Periasamy, Vadiraj Kurdekar, Subbarao Jasti, ..., Muralidhara Padigaru, Vijay Potluri, Ashok R. Venkitaraman

Correspondence

ashokv@ncbs.res.in

In Brief

Periasamy et al. report the development of Bractoppin, a drug-like inhibitor of phosphopeptide recognition by the BRCA1 tBRCT domain, opening avenues to block intracellular signaling via a family of related targets.

Periasamy et al., 2018, Cell Chemical Biology 25, 677–690
June 21, 2018 © 2018 The Authors. Published by Elsevier Ltd.
https://doi.org/10.1016/j.chembiol.2018.02.012

CellPress

CellPress

Targeting Phosphopeptide Recognition by the Human BRCA1 Tandem BRCT Domain to Interrupt BRCA1-Dependent Signaling

Jayaprakash Periasamy,[1,3] Vadiraj Kurdekar,[1,3] Subbarao Jasti,[1,3] Mamatha B. Nijaguna,[1] Sanjana Boggaram,[1] Manjunath A. Hurakadli,[1] Dhruv Raina,[1] Lokavya Meenakshi Kurup,[1] Chetan Chintha,[1] Kavyashree Manjunath,[1] Aneesh Goyal,[1,4] Gayathri Sadasivam,[1,4] Kavitha Bharatham,[1,4] Muralidhara Padigaru,[1,4] Vijay Potluri,[1,4] and Ashok R. Venkitaraman[1,2,4,5,*]
[1]Center for Chemical Biology & Therapeutics, InSTEM, Bellary Road, Bangalore, Karnataka 560065, India
[2]Medical Research Council Cancer Unit, University of Cambridge, Hills Road, Cambridge CB2 0XZ, UK
[3]These authors contributed equally
[4]Senior author
[5]Lead Contact
*Correspondence: ashokv@ncbs.res.in
https://doi.org/10.1016/j.chembiol.2018.02.012

SUMMARY

Intracellular signals triggered by DNA breakage flow through proteins containing BRCT (BRCA1 C-terminal) domains. This family, comprising 23 conserved phosphopeptide-binding modules in man, is inaccessible to small-molecule chemical inhibitors. Here, we develop Bractoppin, a drug-like inhibitor of phosphopeptide recognition by the human BRCA1 tandem (t)BRCT domain, which selectively inhibits substrate binding with nanomolar potency *in vitro*. Structure-activity exploration suggests that Bractoppin engages BRCA1 tBRCT residues recognizing pSer in the consensus motif, pSer-Pro-Thr-Phe, plus an abutting hydrophobic pocket that is distinct in structurally related BRCT domains, conferring selectivity. In cells, Bractoppin inhibits substrate recognition detected by Förster resonance energy transfer, and diminishes BRCA1 recruitment to DNA breaks, in turn suppressing damage-induced G2 arrest and assembly of the recombinase, RAD51. But damage-induced MDC1 recruitment, single-stranded DNA (ssDNA) generation, and TOPBP1 recruitment remain unaffected. Thus, an inhibitor of phosphopeptide recognition selectively interrupts BRCA1 tBRCT-dependent signals evoked by DNA damage.

INTRODUCTION

BRCT domains, first described as a discrete structural motif encoded in the C-terminal region of the breast and ovarian cancer suppressor protein, BRCA1, represent a widely distributed family (PFAM PF00533) of modules that mediate protein-protein interactions involved in the recognition of phosphopeptides (Bork et al., 1997; Koonin et al., 1996; Manke et al., 2003; Yu et al., 2003). BRCT domains with evolutionarily conserved sequences have been identified across all kingdoms of life, where they form functionally critical elements of proteins, which participate in the signaling pathways that preserve genome integrity, through functions in DNA replication and repair (reviewed in Gerloff et al., 2012; Leung and Glover, 2011; Mesquita et al., 2010). While the human proteome encodes 23 different BRCT-containing proteins (Woods et al., 2012), over 245 have been identified in other species.

Twelve of the human proteins containing BRCT domains incorporate more than one copy of the ~100-residue BRCT domain fold, comprising a four-stranded β sheet juxtaposed to three α-helical regions in the order βαββαβα (Williams et al., 2001). In human BRCA1, two BRCT domain folds pack tightly to one another in a head-to-tail orientation, illustrating a subgroup of tandem BRCT (tBRCT) domains that includes the tBRCT domains of the human proteins DNA topoisomerase 2-binding protein 1 (TOPBP1), mediator of DNA damage checkpoint 1 (MDC1) and microcephalin 1 (MCPH1) (reviewed in Gerloff et al., 2012; Leung and Glover, 2011; Mesquita et al., 2010). The BRCA1 tBRCT domain binds phosphorylated peptides with the consensus sequence, pSer-Pro-Thr-Phe, wherein the first and last residues are highly conserved, but amino acid representation in the intervening residues exhibits less stringency (i.e., pSer-X-X-Phe) (Manke et al., 2003; Rodriguez et al., 2003; Yu et al., 2003). Structural analysis of the complex between BRCA1 tBRCT and the consensus phosphopeptide from the DNA helicase, BTB domain and CNC homolog 1 (BACH1) reveals that the phosphopeptide sits in a cleft formed between the tightly packed tBRCT folds. While the conserved pSer residue contacts the polar side chains of Ser1655 and Lys1702 from just one BRCT fold, the Phe residue engages a hydrophobic pocket formed by the side chains of Met1775, Phe1704, Arg1699, and Leu1839 from both of them (Clapperton et al., 2004; Shiozaki et al., 2004; Williams et al., 2004). These contacts "anchor" the phosphopeptide within the binding cleft.

Genetic and biochemical evidence implicates the human BRCA1 tBRCT domain in the recruitment of BRCA1 to cellular sites of DNA damage marked by phosphorylated (γ) histone H2AX, via an interaction of the tBRCT domain with a pSer residue on the adaptor protein ABRAXAS (Wang et al., 2007). In turn,

This is an open access article under the CC BY license (http://creativecommons.org/licenses/by/4.0/).

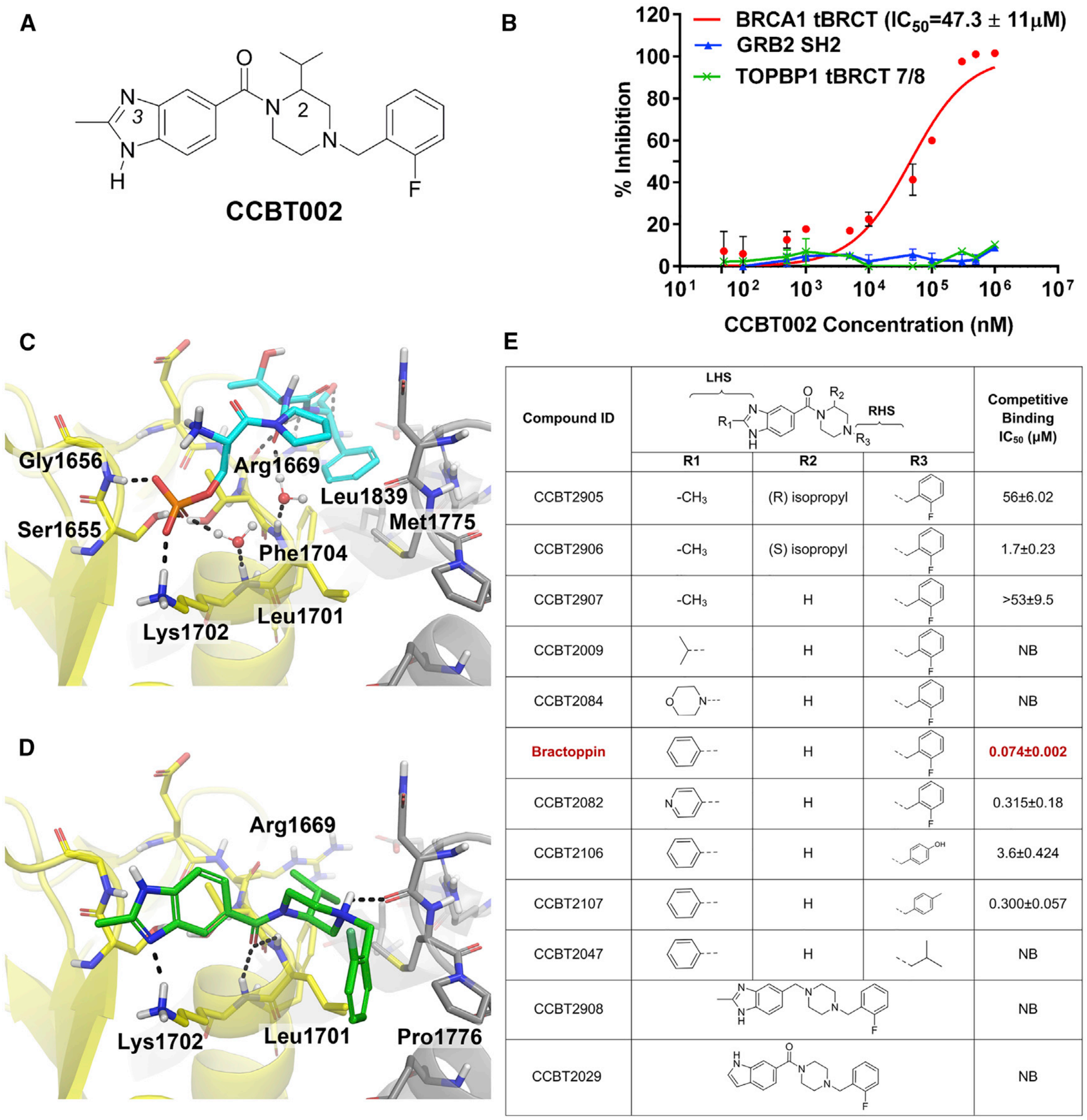

Compound ID	LHS / RHS R1	R2	R3	Competitive Binding IC_{50} (μM)
CCBT2905	$-CH_3$	(R) isopropyl		56±6.02
CCBT2906	$-CH_3$	(S) isopropyl		1.7±0.23
CCBT2907	$-CH_3$	H		>53±9.5
CCBT2009		H		NB
CCBT2084		H		NB
**Bractoppin**		H		**0.074±0.002**
CCBT2082		H		0.315±0.18
CCBT2106		H		3.6±0.424
CCBT2107		H		0.300±0.057
CCBT2047		H		NB
CCBT2908				NB
CCBT2029				NB

Figure 1. Structure-Activity Relationships of Compounds Inhibiting Phosphopeptide Recognition by the BRCA1 tBRCT

(A) Structure of CCBT002, an inhibitor of phosphopeptide recognition by the BRCA1 tBRCT, identified by screening a 128,000-element compound library.

(B) Dose-response and selectivity profile for CCBT002. The compound's ability to inhibit the recognition of cognate biotinylated phosphopeptides to the BRCA1 tBRCT, TOPBP1 tBRCT 7/8, or GRB2 SH2 proteins was measured using an AlphaScreen assay. Percent inhibition is plotted against compound concentration.

(C) Structure of a BACH1 phosphopeptide (cyan sticks) bound to BRCA1 tBRCT (PDB: 3KOK). Yellow or gray shading marks each of the tBRCT modules. Water molecules are red spheres.

(D) Predicted binding mode of CCBT002 (green sticks) in BRCA1 tBRCT. Dotted lines mark hydrogen bonds.

(E) Chemical modifications testing the predicted structure-activity relationships of CCBT002. Structures of CCBT002 analogs are shown in the top panel, with substitutions made at positions R1, R2, or R3 in the central column. The final column shows the IC_{50} value for each compound to competitively inhibit the binding of BACH1 phosphopeptide to BRCA1 tBRCT as measured by MST (NB, no binding). Bractoppin (IC_{50} = 0.074 μM) is marked in red. An inactive analog, CCBT2047, exhibits no binding detectable in this assay. Experiments show the mean ± SD of three independent experiments.

BRCA1 recruitment to these damage sites allows the assembly of a macromolecular complex nucleated around BRCA1, which mediates multiple intracellular signals that choreograph events during the DNA damage response (DDR). BRCA1 coordinates three key limbs of the DDR by engaging the claspin-CHK1 complex to activate the G2 cell-cycle checkpoint for DNA damage (Kumagai and Dunphy, 2000; Sato et al., 2012; Yarden et al., 2002), an endonuclease complex containing CtIP that resects double-stranded DNA dsDNA) to generate overhanging ssDNA substrates (You et al., 2009; Yu et al., 1998), and a DNA recombination complex containing the recombinase RAD51 to localize it to sites of DNA breakage (Bhattacharyya et al., 2000; Scully et al., 1997).

Accordingly, there is considerable current interest in developing selective small-molecule inhibitors that modulate phosphopeptide substrate recognition by the BRCT domain family,

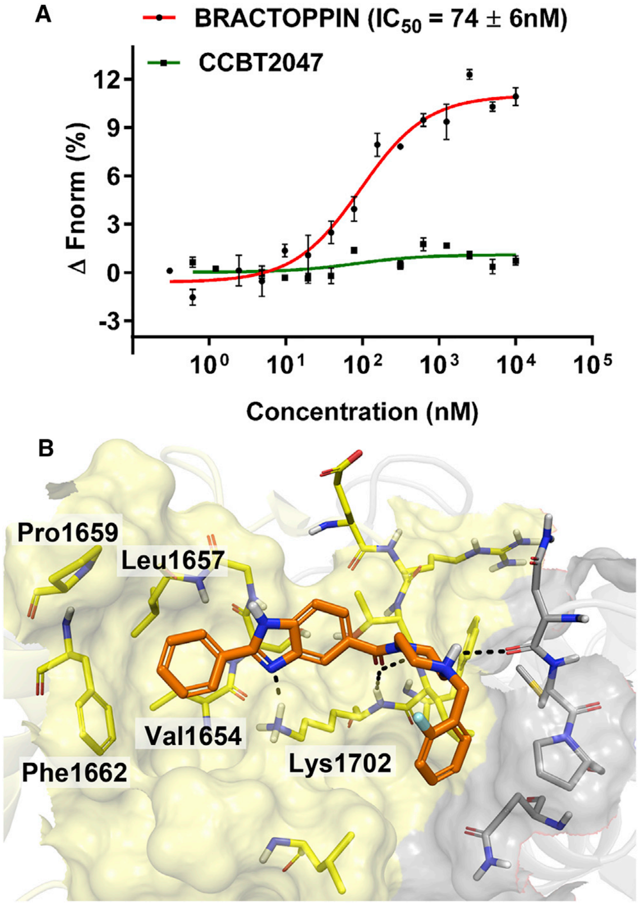

Figure 2. Bractoppin, a Drug-like Inhibitor of Phosphopeptide Recognition by the BRCA1 tBRCT

(A) Competitive inhibition by Bractoppin or its inactive analog CCBT2047 of the binding of BACH1 phosphopeptide to BRCA1 tBRCT measured by MST. Compound concentration in nM is plotted on the x axis, against changes in normalized fluorescence (ΔF_{norm}), on the y axis. Plots represent the mean ± SD (error bars) from three independent experiments. The calculated IC_{50} for Bractoppin is shown.

(B) Predicted binding mode of Bractoppin (orange sticks) in BRCA1 tBRCT (transparent surface). Yellow or gray surface distinguishes each of the BRCT modules. Dotted lines mark hydrogen bonds.

given the essential role in DNA replication and repair played by the proteins containing them. Gossypol, a phenolic natural product with promiscuous biological activities, including male contraception, besides human toxicities, is reported to bind the BRCT domain of poly-ADP ribose polymerase (Na et al., 2015). Peptidic inhibitors of phosphopeptide recognition by the BRCA1 tBRCT (Yuan et al., 2011), including cell-permeant dipeptidic variants incorporating a non-hydrolysable difluoromethylene-substituted phospho-Ser moiety (Na et al., 2014), have been reported. However, their physico-chemical characteristics, and the paucity of information concerning the selectivity of their biological effects, render further development difficult. Thus, the BRCT domain family currently remains inaccessible to selective, drug-like, small-molecule inhibitors.

Here, we report the development of Bractoppin, a drug-like inhibitor of phosphopeptide recognition by the human BRCA1 tBRCT domain. We have explored its structure-activity relationships, exposing contacts that confer activity, as well as its selectivity against structurally related members of the BRCT domain family, suggesting a blueprint for further development. Bractoppin engages its target in cells, inducing characteristic biological effects that discriminate BRCA1-dependent signaling events during the human DDR. Our work provides a template for the future development of drug-like inhibitors against the BRCT domain family, and exemplifies a strategy to selectively modulate intracellular signal transduction by protein kinases by blocking the recognition of their phosphopeptide substrates.

RESULTS

Development and Structure-Activity Relationships of Bractoppin, a Drug-like Inhibitor of Phosphopeptide Binding by BRCA1 tBRCT

We screened a diversity library of 128,000 drug-like molecules (Huggins et al., 2011) using a fluorescence polarization assay (Figures S1A–S1C) to identify compounds that inhibit the binding of the human BRCA1 tBRCT to a TAMRA-labelled phosphopeptide from BACH1. A hit identified in the screen (4-(2-fluorobenzyl)-2-isopropylpiperazin-1-yl) (2-methyl-1H-benzo[d]imidazol-5-yl)methanone, CCBT002 (Figure 1A) is a 394-Da compound that contains no obvious chemical toxicophores or reactive groups, and therefore was regarded as a suitable starting point for development. This compound comprises a 2-isopropyl piperazine core, attached via a carbonyl linkage to benzimidazole ring on nitrogen N1, with an o-fluoro benzyl moiety on nitrogen N4. The enantiomeric form of the isopropyl group in CCBT002 was not established. CCBT002 was validated in a homogeneous AlphaScreen assay (Figure S1D) designed to measure its capacity to displace cognate phosphopeptides either from the human BRCA1 tBRCT, or as controls, from the growth factor receptor-bound protein 2-Src-homology (GRB2-SH2) domain (Nioche et al., 2002) (a pTyr-binding domain structurally distinct from BRCT) or the TOPBP1 tBRCT 7/8 domain (Leung et al., 2011) (structurally a close relative of the BRCA1 tBRCT). CCBT002 competitively inhibits the binding of cognate phosphopeptide to the human BRCA1 tBRCT with a half maximal inhibitory concentration (IC_{50}) of 47.3 μM, but not to the GRB2-SH2 domain or the TOPBP1 tBRCT domain (Figure 1B). These findings suggest that CCBT002 has features of a selective, drug-like inhibitor of phosphopeptide recognition by the BRCA1 tBRCT.

We combined computational chemistry with the synthesis and testing of new compounds to explore and experimentally validate the structure-activity relationships of CCBT002, because its limited solubility in aqueous buffers impeded our attempts to determine its structure bound to the BRCA1 tBRCT using X-ray crystallography. As indicated by prior crystallographic reports (Clapperton et al., 2004; Shiozaki et al., 2004; Williams et al., 2004), substrate binding to BRCA1 tBRCT does not induce major changes in the phosphopeptide-binding site (Figure S1E). Accordingly, we predicted potential binding modes for CCBT002 by molecular docking at the phosphopeptide-binding site (Figure 1C) using Glide (Schrödinger Release, 2015-3, 2015). Different ionization, tautomeric, and chiral states of CCBT002 were considered (Schrödinger Release, 2015-3: LigPrep,

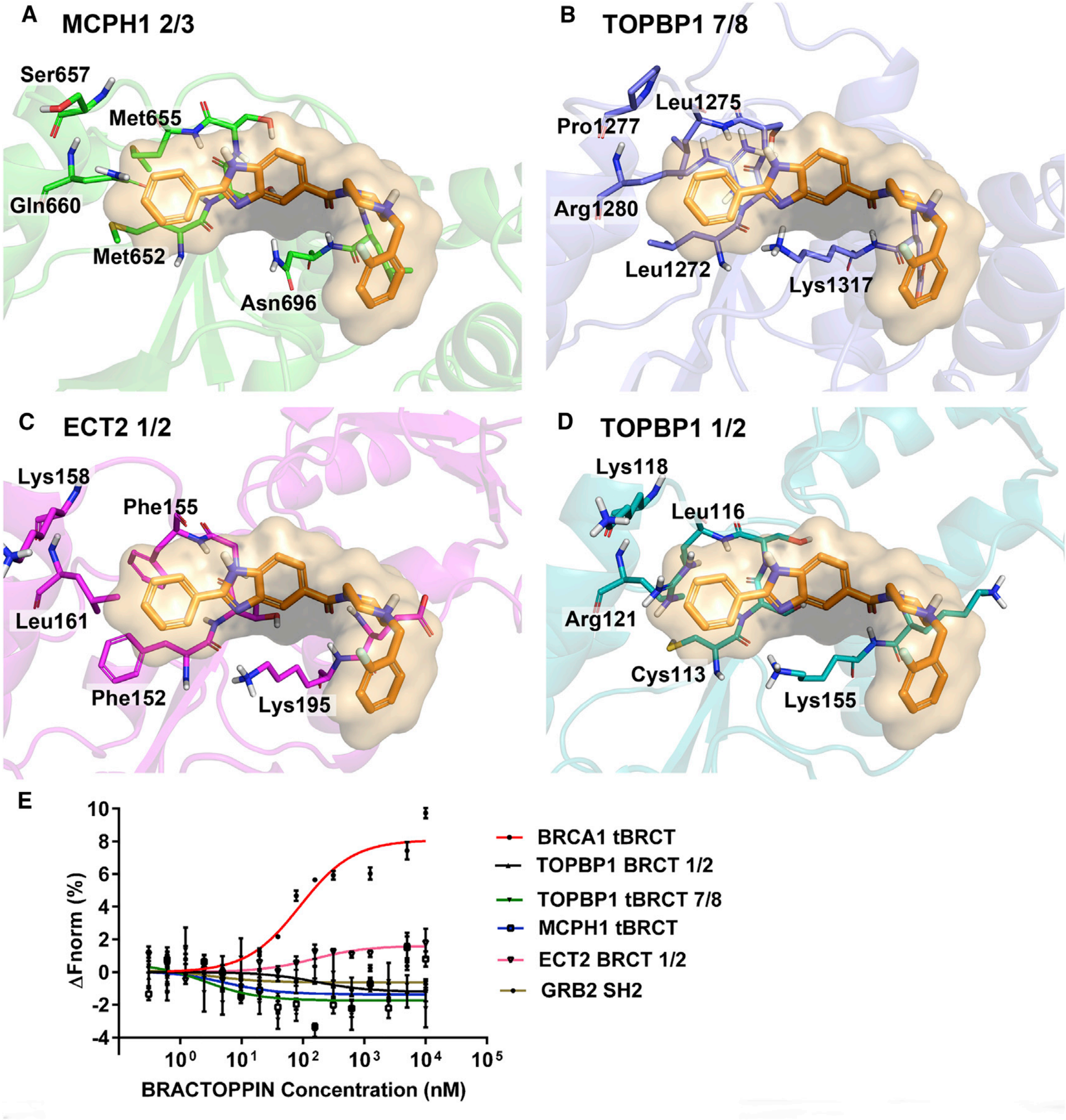

Figure 3. Selectivity of Bractoppin against Structurally Related BRCT Domains

(A–D) Bractoppin (orange) is modeled on the structurally related human BRCT domains of (A) MCPH1 2/3 (PDB: 3SZM), (B) TOPBP1 7/8 (PDB: 3AL3), (C) ECT2 1/2 (PDB: 4N40), and (D) TOPBP1 1/2 (PDB: 3OLC). Each tBRCT domain was aligned to BRCA1 tBRCT's phosphopeptide-binding pocket. Key residues are shown in sticks. Note the differences between these tBRCT domains and BRCA1 tBRCT (Figure 2B) in contacts made by the phenyl ring on the left-hand side of Bractoppin. Also, contacts made by the fluorobenzyl moiety on the right-hand side of Bractoppin are absent in the ECT2 and TOPBP1 1/2 tBRCT domains.

(E) Selectivity profile for Bractoppin as measured by competitive MST assay. Bractoppin concentration in nM is plotted on the x axis, against changes in normalized fluorescence (ΔF_{norm}), on the y axis. The plot shows the mean ± SD of three independent experiments.

2015), yielding several binding poses with similar scores owing to its shallow pocket. Binding modes were evaluated by short, 2-ns molecular dynamics simulations (Bharatham et al., 2017) to identify a stable binding mode (Figure S1F). This predicted binding mode (Figure 1D) shows that CCBT002 recapitulates interactions made in BRCA1 tBRCT by the consensus phosphopeptide pSer-Pro-Thr-Phe, capturing not only polar contacts made in the pSer-recognizing pocket, but, in addition, making a novel contact in a hydrophobic pocket at the interface between the tBRCT folds. Thus, the unsaturated nitrogen, N3 of the benzimidazole core of CCBT002 makes a hydrogen-bonding interaction with the Lys1702 side-chain amine and the carbonyl interacts with the backbone amine group of Lys1702 and Leu1701 in BRCA1 tBRCT. The backbone nitrogens of Leu1701 and Lys1702 act as H-bond donors to contact water molecules observed in several crystal structures of BRCA1 tBRCT (e.g., Leung et al., 2011). The CCBT002 carbonyl group displaces the water while maintaining interactions with these residues. The o-fluoro-benzyl group is partially surrounded by a hydrophobic groove formed between the two BRCT domains, making

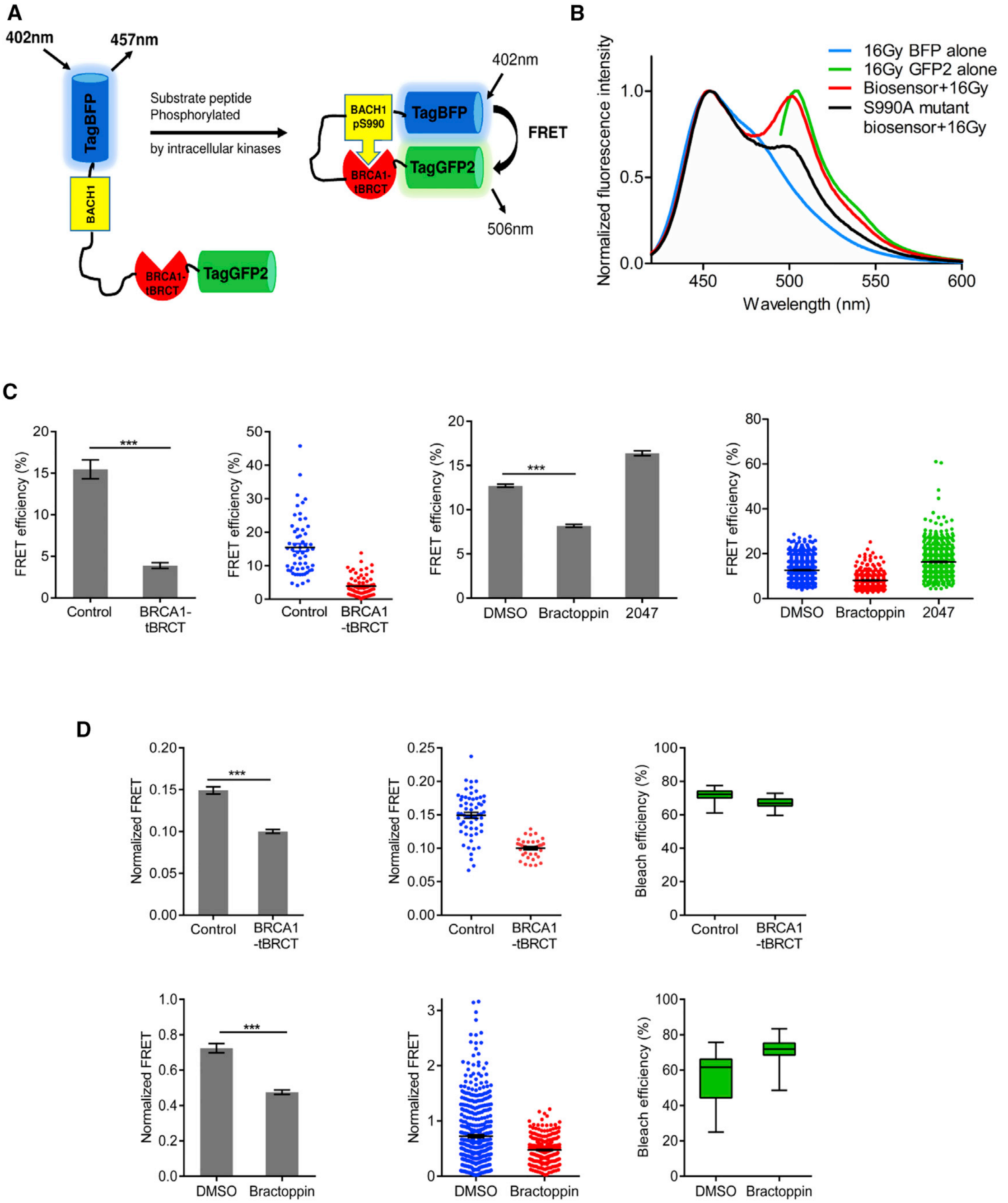

Figure 4. Bractoppin Inhibits Substrate Recognition by the BRCA1 tBRCT in the Cellular Milieu

(A) Schematic depicting the unimolecular FRET biosensor. The BACH1 sequence (yellow box) is phosphorylated by intracellular kinases, triggering engagement by BRCA1 tBRCT (red semi-circle), and inducing FRET by apposition of Tag-BFP and Tag-GFP2 fluorophores. FRET is diminished when compounds or over-expression of BRCA1 tBRCT domain competitively inhibit Tag-BFP phospho-BACH1/Tag-GFP2 BRCA1 tBRCT binding.

(B) Validation of unimolecular FRET biosensor. Fluorescence emission at 420–600 nm wavelengths was measured in HEK293 cells expressing tetracycline (Tet)-inducible biosensor constructs. Constructs encoding Tag-BFP (excited at 402 nm) or Tag-GFP2 (excited at 483 nm) alone were used for spectral correction. Normalized fluorescence intensities are plotted against wavelength. Fluorescence emission is shown for the unimolecular FRET biosensor (red line) or its S990A mutant form (black line) excited at 402 nm 24 hr after the exposure of cells to 16 Gy irradiation.

(C) Effect of Bractoppin or its inactive analog CCBT2047 on FRET measured by sensitized emission. The first panel on the left shows changes in FRET efficiency (mean ± SEM) in biosensor-expressing cells 24 hr after transient transfection with a construct encoding BRCA1 tBRCT, which is expected to competitively inhibit phospho-BACH1/BRCA1 tBRCT binding in the biosensor. The second panel plots FRET efficiency as a dot plot in which each dot represents measurements from a single cell (n = 60, control, or 70, tBRCT). The third and fourth panels show the corresponding FRET measurements after the exposure of cells to 0.5% DMSO (vehicle control), 100 μM Bractoppin, or 100 μM CCBT2047 for 24 hr (n = 640, DMSO; 400, Bractoppin; 650, CCBT2047). Statistical significance was tested using an unpaired, two-tailed t test. ***$p \leq 0.001$.

(legend continued on next page)

contacts with Pro1776, and Leu1701. The S-enantiomer of the isopropyl group on the piperazine ring projects toward the Phe binding pocket making weak hydrophobic interactions with Leu1701 and Met1775 side chains and therefore is predicted to be preferred over the R-enantiomer. To experimentally validate the predicted binding mode, we synthesized compounds (Figure 1E) designed to challenge the expected interactions of CCBT002, and tested their ability to competitively inhibit the binding of a phosphorylated BACH1 substrate peptide to BRCA1 tBRCT using microscale thermophoresis (MST) (Seidel et al., 2013) (Figures S1G and S1H).

CCBT2906, which represents the S-enantiomeric form of the isopropyl group, exhibits stronger activity (IC_{50} = 1.7 μM) than the R-enantiomer (CCBT2905, IC_{50} = 56 μM) or a compound lacking the isopropyl group altogether (CCBT2907, IC_{50} ~53 μM, estimated value owing to non-saturability of binding). Thus, the isopropyl group makes a significant contribution to overall affinity, and its S-enantiomer is preferred, as expected from the predicted binding mode. Removal of the carbonyl group (CCBT2908) renders the compound inactive, consistent with the critical role proposed for this group in contacting the BRCA1 tBRCT residues that recognize pSer. Finally, substitution of the benzimidazole with indole (CCBT2029) reduces affinity, consistent with the predicted role of the benzimidazole nitrogen, N3. Thus collectively, our findings provide evidence to validate the predicted binding mode for CCBT002, and, in particular, to corroborate key contacts made by the benzimidazole nitrogen, N3, and carbonyl with the pSer-recognizing pocket in BRCA1 tBRCT.

To optimize potency, we next explored modifications (Figure 1E) on the left-hand side (LHS) or right-hand side (RHS) of the core structure validated in the experiments above. LHS modifications were designed to capture additional contacts—not made by the cognate phosphopeptide—in the hydrophobic cavity abutting the pSer-recognition pocket that is formed by Phe1662, Leu1657, Val1654, and Pro1659 residues of BRCA1 tBRCT. Substitution of the methyl group (R1) with somewhat bulkier or flexible moieties—either isopropyl (CCBT2009) or morpholinyl (CCBT2084)—abrogated activity. However, introduction of a rigid phenyl group at R1 yielded a compound (Bractoppin) with nanomolar activity (IC_{50} = 0.074 μM).

Bractoppin (Figure 1E) is a 414-Da compound, with nanomolar potency in displacing cognate BACH1 phosphopeptide substrate from the BRCA1 tBRCT as measured by MST (Figure 2A). Its predicted binding mode to BRCA1 tBRCT (Figure 2B) reveals favorable hydrophobic interactions in the hydrophobic cavity, as well as a T-shaped, pi-pi stacking interaction with Phe1662, that together significantly contribute toward its activity. Indeed, substitution of the phenyl ring at R1 in Bractoppin with a 4-pyridyl group (CCBT2082) decreased activity by 5-fold by affecting the stacking interaction with Phe1662.

On the RHS of Bractoppin, the benzene ring of the benzyl group situated at the interface between the two BRCT folds appears critical for activity, because its modification to iso-butyl (CCBT2047) abolishes activity. Substitutions at the para-position of the benzyl ring, including hydroxyl (CCBT2106, IC_{50} = 3.6 μM) or methyl (CCBT2107, IC_{50} = 0.3 μM) groups, reduced activity indicating that the para-position prefers hydrophobic over polar groups, as predicted in the binding mode. Thus, collectively, our findings identify Bractoppin as a potent inhibitor of phosphopeptide recognition by the BRCA1 tBRCT, and provide multiple lines of evidence to support its predicted binding mode and structure-activity relationships.

Selectivity of Bractoppin against Structurally Related tBRCT Domains

The predicted binding mode for Bractoppin in BRCA1 tBRCT suggests a structural rationale for its potential selectivity against other related human tBRCT domains, assuming that the compound binds in a similar orientation at their respective phosphopeptide binding sites. These include the tBRCT domains from MCPH1, TOPBP1, and epithelial cell transforming 2 (ECT2). TOPBP1 contains multiple BRCT modules, of which BRCT7 and BRCT8 are arranged in a tandem head-to-tail array (TOPBP1 7/8) that structurally resembles the BRCA1 tBRCT (Leung and Glover, 2011), as are the tBRCT domains from MCPH1 (Shao et al., 2012; Singh et al., 2012). In contrast, the BRCT folds comprising the tBRCT domains of ECT2 (Zou et al., 2014) or TOPBP1 1/2 (Rappas et al., 2011) are rotated by ~90° compared with the BRCA1 tBRCT, such that they assume a perpendicular orientation to one another.

The phenyl ring decorating the LHS of Bractoppin captures contacts in a hydrophobic cavity formed by BRCA1 tBRCT residues Phe1662, Leu1657, Val1654, and Pro1659 (Figure 2B). This hydrophobic cavity is distinct from other tBRCT domains, including those of MCPH1, TOPBP1 7/8, ECT2, or TOPBP1 1/2, owing to differences in its lining residues (Figures 3A–3D). Moreover, contacts made in BRCA1 tBRCT with the fluorobenzyl moiety on the RHS of Bractoppin are absent in the ECT2 and TOPBP1 1/2 tBRCT domain, owing to changes in the orientation between the BRCT modules compared with BRCA1 tBRCT. Consistent with the predicted structural rationale for selectivity, Bractoppin does not detectably bind to fluorescently labeled tBRCT domains from MCPH1, TOPBP1 7/8, ECT2, or TOPBP1 1/2 as measured by competitive MST (Figure 3E). Thus, collectively, our findings validate and provide a structural rationale for the selectivity of Bractoppin against several related tBRCT domains from human proteins.

Bractoppin Inhibits Substrate Recognition by the BRCA1 tBRCT in Cells

We deployed a genetically encoded, unimolecular biosensor that exploits Förster resonance energy transfer (FRET) to detect intracellular target engagement by Bractoppin. The biosensor comprises the BRCA1 tBRCT, amino N-terminally fused to the fluorophore monomeric GFP (Tag-GFP2), and connected to a blue fluorescent protein (Tag-BFP) via a 16-residue sequence

(D) Effect of Bractoppin or its inactive analog CCBT2047 on FRET measured by acceptor photobleaching. As above, the first panel on the left shows changes in FRET efficiency after transient transfection with BRCA1 tBRCT, or exposure to DMSO or Bractoppin, while the central panel plots FRET efficiency as a dot plot (n = 60, control; 40, tBRCT; 380, DMSO; 370, Bractoppin). FRET efficiency was normalized to bleach efficiency in each experiment (final panel on the right, top and bottom). Statistical significance was tested using an unpaired, two-tailed t test. ***$p \leq 0.001$. Similar results were observed in four independent repeats.

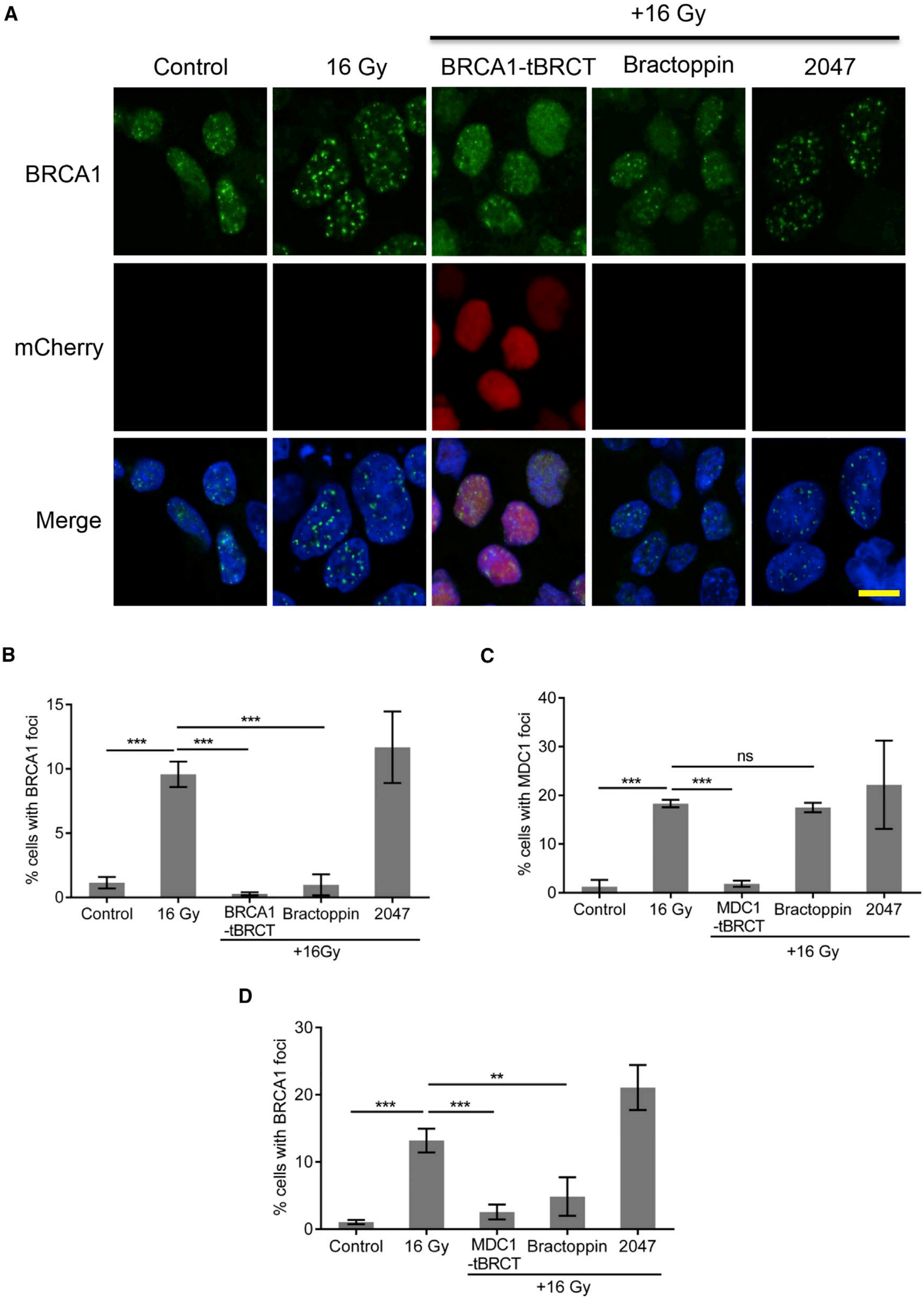

Figure 5. Bractoppin Selectively Inhibits Cellular Substrate Recognition by the tBRCT Domain Family

(A) Confocal images depicting at high magnification (189×) the recruitment of the BRCA1 protein into nuclear foci after the indicated treatments (untreated cells [0 Gy]; irradiation alone [16 Gy]; Tet-induced BRCA1 tBRCT expression 30 hr before irradiation; 100 μM Bractoppin or its inactive analog CCBT2047 added 6 hr after irradiation). Staining 18 hr after irradiation in the upper row is for BRCA1 (green), middle row, for mCherry-BRCA1 tBRCT (red); lower row, merged red and green staining, with DNA staining (DAPI) in blue. Scale bar represents 10 μm.

(legend continued on next page)

from BACH1 (Figure 4A). When this BACH1 sequence is phosphorylated by intracellular protein kinases, it is recognized by the BRCA1 tBRCT, apposing the Tag-BFP and Tag-GFP2 reporters to induce FRET. HEK293 cells were stably transfected with a construct encoding the biosensor under the control of a tetracycline-inducible promoter. Constructs encoding Tag-BFP or Tag-GFP2 alone were used as controls for spectral correction to calculate FRET efficiency. For validation, we first exposed cells stably expressing the biosensor to 16 Gy ionizing radiation (IR), which is reported to activate signals leading to BACH1 phosphorylation (Peng et al., 2006; Shiozaki et al., 2004). Indeed, this suffices to induce FRET (Figure 4B). FRET is abrogated (Figure 4B) by the replacement of the Ser residue in the BACH1 substrate peptide that undergoes phosphorylation, with the non-phosphorylable residue, Ala (Ser990Ala biosensor), confirming the specificity of pSer recognition by the BRCA1 tBRCT in the biosensor construct.

Overexpression of a construct encoding the BRCA1 tBRCT domain—which is expected to mimic the cellular effects of Bractoppin by competitively inhibiting phosphopeptide substrate recognition—suffices to decrease FRET efficiency detected by the biosensor, as measured by sensitized emission (Figure 4C) or acceptor photobleaching (Figure 4D). Notably, Bractoppin–but not its inactive iso-butyl substituted analog, CCBT2047—also inhibits FRET (Figures 4C, 4D, and S2).

Bractoppin Selectively Inhibits Cellular Substrate Recognition by the BRCA1 but Not MDC1 tBRCT

Following exposure to IR, BRCA1 protein is recruited to cellular sites of DNA damage, where it assembles in microscopic foci, through the recognition of a phosphorylated motif in the adaptor protein ABRAXAS via the BRCA1 tBRCT (Wang et al., 2007; Wu et al., 2016). Again, overexpression of the BRCA1 tBRCT domain, but neither the tBRCT M1775R nor S1655A/K1702M mutant forms deficient in phosphopeptide substrate binding, decreases BRCA1 foci formation following DNA damage as measured by high-content microscopy using a murine monoclonal antibody against BRCA1 (Figures S3A–S3C). Bractoppin, but not its inactive analog, CCBT2047, also inhibits the formation of radiation-induced BRCA1 foci (Figures 5A and 5B). Similar results were observed in a different cell line using an alternative monoclonal antibody directed against a distinct BRCA1 epitope (Figure S3D).

The human protein MDC1 is also recruited to microscopic foci formed at sites of DNA damage through interactions mediated by its tBRCT domains (Lou et al., 2003; Stewart et al., 2003; Stucki et al., 2005). While the MDC1 tBRCT domain is structurally related to that of BRCA1 (Campbell et al., 2010), the extent of substrate cross-recognition by these tBRCT domain family members remains unclear. In turn, given the potential for substrate cross-recognition in the cellular milieu, whether drug-like inhibitors of substrate recognition by tBRCT can elicit selective biological effects is uncertain. To test this issue, we first determined the effects of MDC1 tBRCT overexpression on the formation of damage-induced MDC1 or BRCA1 foci in cells (Figures 5C and 5D). As expected, MDC1 tBRCT suppresses both MDC1 and BRCA1 foci formation, since MDC1 recruitment to damage sites precedes and is required for BRCA1 accumulation (Huen et al., 2007; Kolas et al., 2007; Mailand et al., 2007). Interestingly, however, Bractoppin, but not its inactive analog CCBT2047, selectively inhibits damage-induced BRCA1 foci formation, but has little effect on the radiation-induced accumulation of MDC1 at sites of DNA damage (Figures 5C, 5D, and S3E). Similarly, Bractoppin has little effect on the radiation-induced recruitment of TOPBP1 (Figure S3F), a protein containing multiple, structurally related tBRCT domains, again speaking to the selectivity of its effects. Thus, collectively, our observations provide multiple lines of evidence that Bractoppin selectively inhibits the recognition of phosphopeptide substrates by the human BRCA1 tBRCT, suppressing the recruitment of BRCA1, but not other proteins containing structurally related tBRCT domains, to cellular DNA damage sites.

Bractoppin Interrupts DNA Damage Signaling for G2 Arrest

BRCA1 recruitment to sites of DNA damage initiates events leading to cell-cycle arrest at the G2 checkpoint (Yarden et al., 2002; Yu and Chen, 2004). Overexpression of the BRCA1 tBRCT inhibits damage-induced G2 arrest (Figures 6A and 6B), whereas the single (M1775R) and the double (S1655A, K1702M) tBRCT mutants do not (Figures S4A–S4D). G2 arrest is also inhibited in a dose-dependent manner by Bractopppin, but not by its inactive analog CCBT2047 (Figures 6A and 6B), suggesting that the compound interrupts signals that activate the G2 checkpoint. Failure to engage the G2 checkpoint sensitizes cells to the cytotoxic effects of IR (Tenzer and Pruschy, 2003). Indeed, overexpression of the BRCA1 tBRCT significantly enhances cytotoxicity induced by exposure to 1 Gy IR, as does treatment with Bractoppin in a dose-dependent manner (Figure 6C). Together, these findings demonstrate that Bractoppin inhibits intracellular signals essential for the response of human cells to DNA damage.

Bractoppin Discriminates BRCA1-Dependent Steps in DNA Repair by Homologous Recombination

IR-induced double-strand DNA breaks are repaired in dividing cells by homologous DNA recombination (HR), a mechanism in which BRCA1, and the related tumor suppressor protein BRCA2, have been implicated at several steps (reviewed in Venkitaraman, 2014). HR is initiated by the resection of DNA ends to generate ssDNA tracts that are coated by the ssDNA-binding

(B) Percentage of cells positive for radiation-induced nuclear BRCA1 foci (mean ± SD; n = 15,000, 0 Gy, 20,000, 16 Gy; 10,500, BRCA1 tBRCT; 10,600, Bractoppin; 13,000, CCBT2047) enumerated by high-content imaging at low magnification (see the STAR Methods). Treatment conditions were as described in (A). Statistical significance was determined using an unpaired two-tailed t test. ***$p \leq 0.001$. Similar results were observed in three independent repeats.

(C) Percentage of cells positive for radiation-induced nuclear MDC1 foci (mean ± SD; n = 24,000, 0 Gy; 6,000, 16 Gy; 14,500, MDC1-tBRCT; 8,000, Bractoppin; 4,600, CCBT2047) enumerated as above. Treatment conditions were as described in (A), except that the effect of Tet-induced MDC1 tBRCT expression was tested. Statistical significance was determined using an unpaired two-tailed t test. ***$p \leq 0.001$; ns, not significant.

(D) Cells treated as in (C) were stained for nuclear BRCA1 foci. Similar results were observed in three independent repeats. **$p \leq 0.01$; ***$p \leq 0.001$.

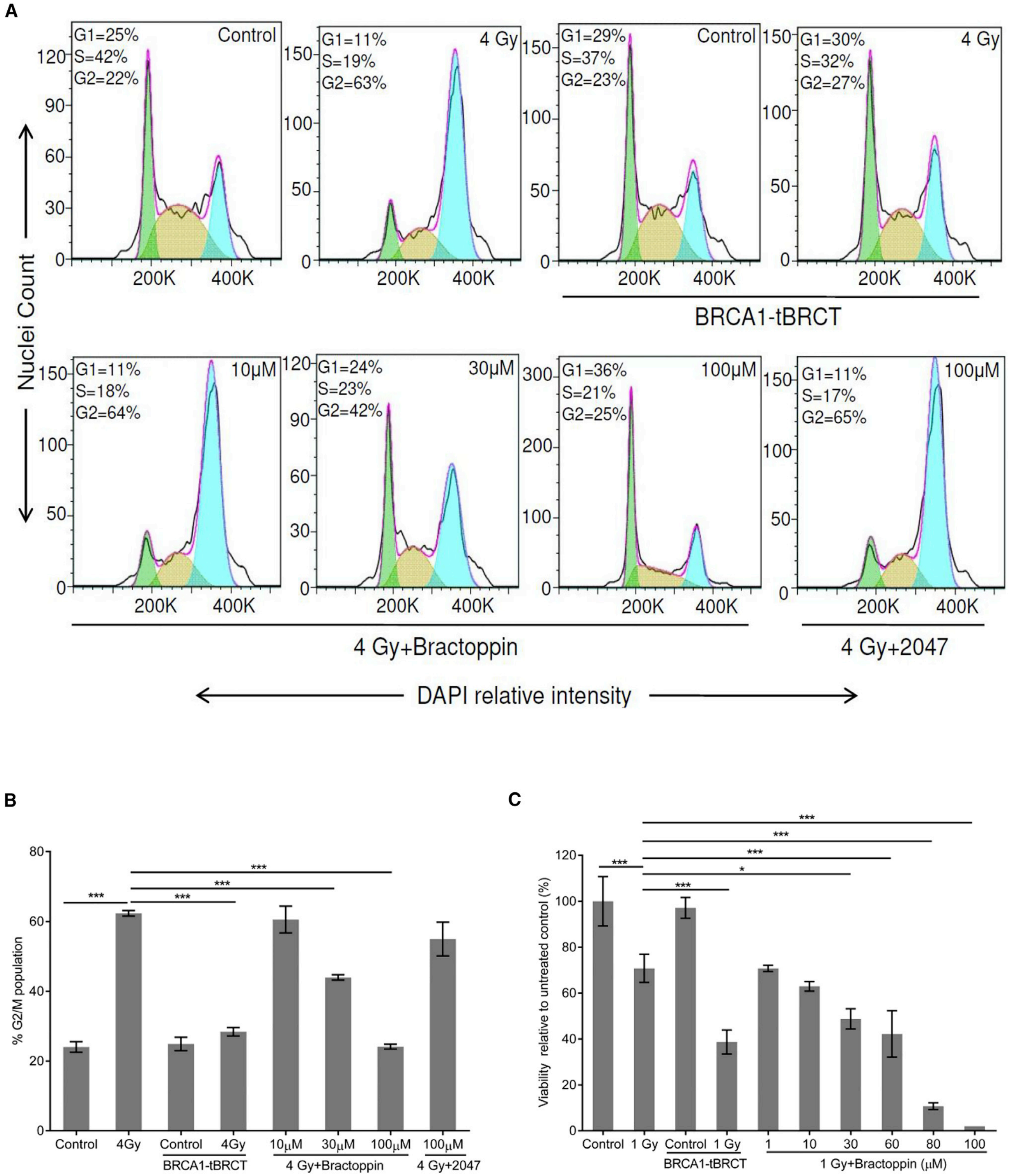

Figure 6. Bractoppin Interrupts DNA Damage Signaling for G2 Arrest

(A) Representative histograms of cell-cycle distribution measured by flow cytometry after DAPI staining. Cells were irradiated with 4 Gy at 8 hr after synchronous release into the cell cycle from thymidine block, and measurements made 16 hr later. The histograms show unirradiated cells (Control), or cells exposed to 4 Gy, with or without additional treatments using Tet-inducible BRCA1 tBRCT expression, 10–100 μM Bractoppin or 100 μM CCBT2047. Tet-induced BRCA1 tBRCT expression was for 32 hr before radiation, while compounds were added 0.5 hr before. A total of 15,000 cells were analyzed per condition, in replicates of 3 (green, G1, yellow, S, blue, G2/M).

(legend continued on next page)

factor, replication protein A (RPA32) (reviewed in Symington and Gautier, 2011). These ssDNA tracts not only stimulate the activation of the G2 checkpoint via the ATR-ATRIP complex (Kumagai et al., 2006; Zou and Elledge, 2003), but also serve as substrates for the formation of ordered nucleoprotein assemblies containing the recombination enzyme, RAD51 (reviewed in San Filippo et al., 2008). While genetic studies suggest that BRCA1 is essential to signal G2 arrest, and recruit RAD51 to DNA damage sites, DNA end-resection and the accumulation of RPA can proceed through BRCA1-dependent as well as BRCA1-independent mechanisms (Cruz-García et al., 2014; Polato et al., 2014; Reczek et al., 2013). Indeed, Bractoppin selectively suppresses IR-induced RAD51 foci, but has little effect on RPA32 accumulation (Figures 7A–7D), confirming that it discriminates BRCA1-dependent from BRCA1-independent steps leading to HR. Moreover, these findings separate an unappreciated requirement for substrate recognition by the BRCA1 tBRCT in DNA end-resection and ssDNA generation at damage sites, from the events that trigger RAD51 recruitment to those sites, with implications for the biology of BRCA1 as well as the development of selective chemical modulators of its function.

DISCUSSION

Intracellular signaling cascades initiated by protein kinases are critical to cellular physiology, and often perturbed in human diseases. ATP-competitive inhibitors of protein kinase activity that are currently used to modulate these signaling cascades for mechanistic analysis or disease therapy frequently induce pleiotropic effects by suppressing multiple pathways downstream of the inhibited enzyme. The work we report here exemplifies an alternative strategy to selectively interrupt kinase signaling cascades, by blocking the protein-protein interactions between phosphorylated protein substrates and their cognate recognition domains that propagate kinase-initiated signals. In particular, we have identified Bractoppin, a drug-like chemical inhibitor of phosphopeptide substrate recognition by the human BRCA1 tBRCT domain. Our findings have several important implications.

The BRCA1 tBRCT domain represents a prominent member of a vital family comprising >200 phosphopeptide-binding domains that mediate kinase-initiated signaling pathways from bacteria to humans, but remain refractory to selective, non-peptidic inhibitors despite much recent effort. By characterizing and experimentally validating a structural model for the interaction between Bractoppin and the BRCA1 tBRCT, our findings open avenues to target other members of this family. We provide evidence that Bractoppin not only engages residues in BRCA1 tBRCT that recognize the phosphopeptide substrate, but also occupies two additional hydrophobic pockets that are not explored by the phosphopeptide. These hydrophobic pockets are absent in structurally related tBRCT domains such as those of MCPH1 or MDC1. Moreover, other structurally related tBRCT domains found in ECT2 or TOPBP1 1/2 exhibit an altered orientation between the two individual BRCT modules that occludes Bractoppin binding. Compounds designed to validate the structure-activity relationships of Bractoppin provide plausible starting points from which to identify inhibitors that engage these related domains. Together, our findings suggest a structural rationale for the experimentally observed *in vitro* selectivity of Bractoppin for BRCA1 tBRCT, and also provide a blueprint for the design of new inhibitors selective for other members of the BRCT domain family.

Several lines of evidence indicate that Bractoppin selectively inhibits in the cellular milieu substrate recognition by BRCA1 tBRCT. Bractoppin engages its target in cells as detected by an unimolecular FRET biosensor designed to report inhibition of BACH1 phosphopeptide recognition. It selectively inhibits the recruitment of BRCA1 protein to cellular sites of DNA damage, an event mediated by the protein-protein interactions of the BRCA1 tBRCT domain. But it has little effect on the recruitment of MDC1 or TOPBP1 via their structurally related tBRCT domains.

The selectivity of Bractoppin's effects is further attested by the phenotypes triggered by overexpression of the BRCA1 or MDC1 tBRCT domains, which are predicted to mimic the action of chemical inhibitors by competitively suppressing the protein-protein interactions of endogenous BRCA1 or MDC1. While BRCA1 tBRCT overexpression suppresses BRCA1 recruitment to damage-induced foci, MDC1 tBRCT overexpression diminishes both MDC1 and BRCA1 recruitment. Our results speak not only to the biological selectivity of substrate recognition via members of the tBRCT domain family, but also the potential to modulate intracellular signaling with equivalent precision through the development of selective inhibitors.

Of note, however, Bractoppin exhibits nanomolar on-target potency *in vitro*, but elicits cellular effects only at 10–100 μM, suggesting that compound exposure may be variously limited by uptake, stability or metabolism in different cell types. This observation highlights limitations to be addressed by future chemical optimization and biological studies.

Human BRCA1 normally plays a vital tumor suppressive role by acting as the hub of a macromolecular assembly formed on damaged DNA, which transmits intracellular signals to activate the G2 cell-cycle checkpoint, and also to regulate reactions that lead to the error-free repair of damaged DNA by RAD51-mediated HR. HR is initiated by the endonucleolytic resection of broken dsDNA ends into overhanging ssDNA, which involves BRCA1 tBRCT-dependent macromolecular complexes containing the

(B) The percentage of cells with 4N DNA content representing G2/M phases of the cell cycle corresponding to the treatment conditions as in (A). Results are representative of three independent repeats. Statistical significance was tested using Dunnett's multiple comparisons test, post one-way ANOVA. ***$p \leq 0.001$.
(C) Percentage of viable cells relative to untreated controls measured using calcein AM dye and cell counting (mean ± SD, n = 3). Cells were irradiated with 1 Gy, and viability was measured 7 days later. The bars show unirradiated cells (Control), or cells exposed to 1 Gy, without (bars 1, 2), or with additional treatments using Tet-inducible BRCA1 tBRCT expression (bars 3, 4), or 1–100 μM Bractoppin (bars 5–10). Tet-induced BRCA1 tBRCT expression was for 6 hr before radiation, while compounds were added 1 hr before. Results are representative of three independent repeats. Statistical analysis was done using Dunnett's multiple comparisons test post one-way ANOVA. *$p \leq 0.05$; ***$p \leq 0.001$.
Error bars represent standard deviation from the mean.

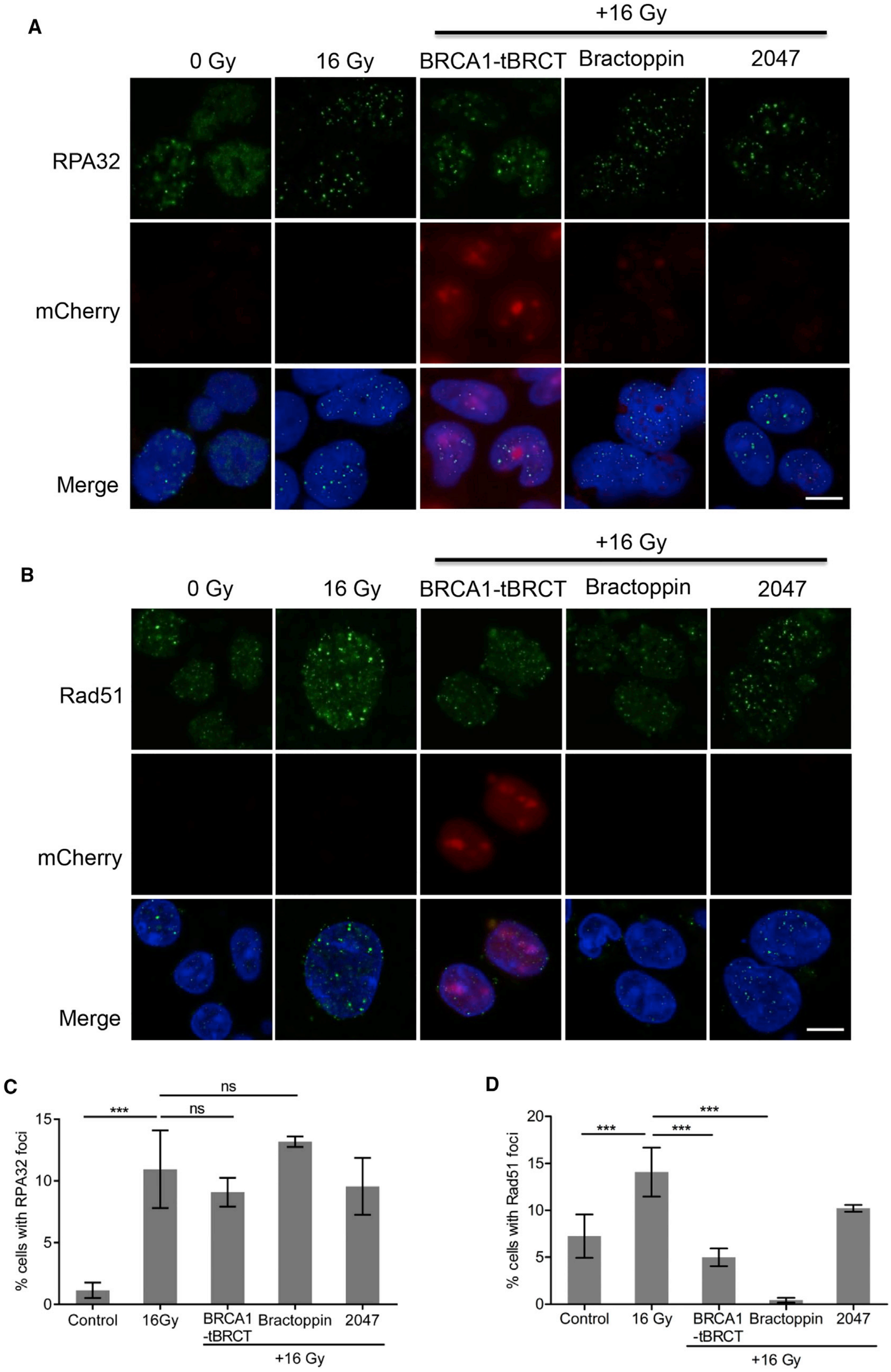

(legend on next page)

CtIP protein. However, BRCA1 is not indispensable for ssDNA generation at dsDNA breaks owing to the existence of alternative, BRCA1-independent mechanisms. In contrast, BRCA1 inactivation diminishes the assembly of the RAD51 recombination enzyme on ssDNA substrates generated by end-resection, likely through its ability to form complexes with the HR mediators PALB2 and BRCA2 (reviewed in San Filippo et al., 2008; Symington and Gautier, 2011; Venkitaraman, 2014). Indeed, while Bractoppin suppresses the enforcement of the G2 checkpoint for DNA damage, as well as the assembly of RAD51 at damage sites, it spares ssDNA generation marked by the ssDNA-binding protein, RPA32. Thus, the effects of Bractoppin on signals evoked by DNA damage endorse its selectivity for BRCA1-dependent steps.

Diminished RAD51 foci formation at sites of DNA damage typically signifies the suppression of DNA repair by HR (reviewed in San Filippo et al., 2008), suggesting that Bractoppin inhibits this repair mechanism. BRCA1 is, however, implicated in multiple events during the sensing, signaling, and repair of different forms of DNA damage, warranting future characterization of the effects of Bractoppin on HR and other mechanisms for DNA repair.

Our results collectively provide proof-of-concept for a strategy to selectively interrupt intracellular signaling pathways initiated by protein kinases using drugs that block the molecular recognition of phosphorylated proteins. The recent finding that inhibitors of the protein-protein interactions of the phosphopeptide-binding Polo-box domains of Polo-like kinases can be used to target *KRAS* mutant cancers (Narvaez et al., 2017) speaks to the future therapeutic potential for such a strategy.

Our finding that Bractoppin enhances the cell-killing effects of IR suggests one such therapeutic avenue. The majority of patients with solid tumors receive therapeutic radiation, but tumor recrudescence and off-target effects remain major clinical problems. Radiation-sensitizing agents may alleviate such issues by decreasing the radiation dosage required for total tumor clearance. In addition, it has also been suggested that inhibitors of BRCA1 may sensitize tumor cells to the effect of targeted therapies such as PARP1 inhibitors. But because the systemic administration of BRCA1 inhibitors combined with PARP1 inhibitors is likely to induce PARP1 inhibitor sensitivity even in normal tissues, we are skeptical about the therapeutic index of such an approach. Aside from cancer therapy using BRCA1 tBRCT inhibitors, however, the potential utility of selective tBRCT inhibitors in the treatment of other diseases remains relatively under-explored. For example, the development of selective targeting tBRCT domains in bacterial proteins that mediate DNA replication or genome maintenance may open potential applications in the treatment of infections. The work we report here represents an initial step to the future exploration of such strategies.

SIGNIFICANCE

The development of Bractoppin exemplifies a strategy to chemically inhibit phosphopeptide substrate recognition by BRCT domains, evolutionarily conserved mediators of genome maintenance pathways from prokaryotes to eukaryotes. The structure-activity relationships of Bractoppin open avenues to selectively target other members of this domain family, which are attractive, but currently inaccessible, targets for drug discovery against human diseases. Unlike ATP-competitive inhibitors of DNA damage-activated protein kinases, Bractoppin preferentially inhibits BRCA1 tBRCT-dependent steps in the DNA damage response. Thus, our work illustrates a new approach to selectively interrupt intracellular signaling pathways initiated by protein kinases using drugs that block the molecular recognition of phosphorylated proteins.

STAR★METHODS

Detailed methods are provided in the online version of this paper and include the following:

- KEY RESOURCES TABLE
- CONTACT FOR REAGENT AND RESOURCE SHARING
- EXPERIMENTAL MODEL AND SUBJECT DETAILS
  - Compounds
  - Antibodies
  - Expression Constructs
  - Cell Lines and Cell Culture
  - Cell Irradiation
- METHOD DETAILS
  - Chemical Synthesis
  - Molecular Docking
  - Molecular Dynamic (MD) Simulations
  - Protein Expression and Purification
  - Fluorescence Polarization (FP) Assay
  - Alpha Screen Assay
  - Microscale Thermophoresis (MST)
  - Unimolecular FRET Sensor Assay
  - Immunofluorescence Staining for Damage-Induced Foci
  - Cell Cycle Profiles
  - Cell Viability Assays
- QUANTIFICATION AND STATISTICAL ANALYSIS

Figure 7. Bractoppin Selectively Interrupts BRCA1-Dependent Steps in DNA Repair by Homologous Recombination
(A) Confocal images depicting at high magnification the recruitment of the RPA32 protein into nuclear foci after the indicated treatments. Experiments were carried out as described as in Figure 5A. Staining in the upper row is for RPA32 (green), middle row, for mCherry-BRCA1 tBRCT (red); lower row, merged red and green staining, with DNA staining (DAPI) in blue. Scale bar represents 10 μm.
(B) Recruitment of RAD51 protein into nuclear foci, measured and depicted as described in (A). Scale bar represents 10 μm.
(C) Percentage of cells positive for radiation-induced nuclear RPA32 foci (mean ± SD; n = 5,300, 0 Gy; 3,200, 16 Gy; 3,600, BRCA1 tBRCT; 3,400, Bractoppin; 3,500, CCBT2047) enumerated by high-content imaging (see the STAR Methods). Statistical significance was performed using an unpaired two-tailed t test. ***$p \leq 0.001$. Similar results were observed in three independent repeats.
(D) Percentage of cells containing nuclear RAD51 foci enumerated and depicted as described in (B). ***$p \leq 0.001$.

SUPPLEMENTAL INFORMATION

Supplemental Information includes four figures and can be found with this article online at https://doi.org/10.1016/j.chembiol.2018.02.012.

ACKNOWLEDGMENTS

The work reported here was funded by the Department of Biotechnology, Government of India, at the Center for Chemical Biology & Therapeutics; sanction order no. BT/PR7222/MED/31/1901/2012, dated 11.01.2013. A.R.V.'s primary appointment is at the University of Cambridge, where his work was also supported by UK Medical Research Council award MR/N501876/1.

AUTHOR CONTRIBUTIONS

Methodology, Resources, Investigation, Formal Analysis, Visualization, J.P., V.K., S.J., M.B.N., S.B., M.A.H., D.R., L.M.K., C.C., K.M., A.G., G.S., K.B., M.P., V.P., and A.R.V.; Validation, Data Curation, Visualization, Writing – Review & Editing, G.S., K.B., V.P., M.P., A.G., and A.R.V.; Conceptualization, Writing – Original Draft, Review & Editing, Supervision, Project Administration, Funding Acquisition, A.R.V.

DECLARATION OF INTERESTS

The authors declare no competing interests.

Received: September 6, 2017
Revised: November 24, 2017
Accepted: February 22, 2018
Published: March 29, 2018

REFERENCES

Bharatham, N., Finch, K.E., Min, J., Mayasundari, A., Dyer, M.A., Guy, R.K., and Bashford, D. (2017). Performance of a docking/molecular dynamics protocol for virtual screening of nutlin-class inhibitors of Mdmx. J. Mol. Graph. Model. *74*, 54–60.

Bhattacharyya, A., Ear, U.S., Koller, B.H., Weichselbaum, R.R., and Bishop, D.K. (2000). The breast cancer susceptibility gene BRCA1 is required for subnuclear assembly of Rad51 and survival following treatment with the DNA cross-linking agent cisplatin. J. Biol. Chem. *275*, 23899–23903.

Bork, P., Hofmann, K., Bucher, P., Neuwald, A.F., Altschul, S.F., and Koonin, E.V. (1997). A superfamily of conserved domains in DNA damage-responsive cell cycle checkpoint proteins. FASEB J. *11*, 68–76.

Campbell, S.J., Edwards, R.A., and Glover, J.N. (2010). Comparison of the structures and peptide binding specificities of the BRCT domains of MDC1 and BRCA1. Structure *18*, 167–176.

Capes-Davis, A., Reid, Y.A., Kline, M.C., Storts, D.R., Strauss, E., Dirks, W.G., Drexler, H.G., MacLeod, R.A., Sykes, G., Kohara, A., and Nakamura, Y. (2013). Match criteria for human cell line authentication: where do we draw the line? Int. J. Cancer *132*, 2510–2519.

Clapperton, J.A., Manke, I.A., Lowery, D.M., Ho, T., Haire, L.F., Yaffe, M.B., and Smerdon, S.J. (2004). Structure and mechanism of BRCA1 BRCT domain recognition of phosphorylated BACH1 with implications for cancer. Nat. Struct. Mol. Biol. *11*, 512–518.

Cruz-García, A., López-Saavedra, A., and Huertas, P. (2014). BRCA1 accelerates CtIP-mediated DNA-end resection. Cell Rep. *9*, 451–459.

Gerloff, D.L., Woods, N.T., Farago, A.A., and Monteiro, A.N. (2012). BRCT domains: a little more than kin, and less than kind. FEBS Lett. *586*, 2711–2716.

Gorringe, K.L., Chin, S.F., Pharoah, P., Staines, J.M., Oliveira, C., Edwards, P.A., and Caldas, C. (2005). Evidence that both genetic instability and selection contribute to the accumulation of chromosome alterations in cancer. Carcinogenesis *26*, 923–930.

Huen, M.S., Grant, R., Manke, I., Minn, K., Yu, X., Yaffe, M.B., and Chen, J. (2007). RNF8 transduces the DNA-damage signal via histone ubiquitylation and checkpoint protein assembly. Cell *131*, 901–914.

Huggins, D.J., Venkitaraman, A.R., and Spring, D.R. (2011). Rational methods for the selection of diverse screening compounds. ACS Chem. Biol. *6*, 208–217.

Kolas, N.K., Chapman, J.R., Nakada, S., Ylanko, J., Chahwan, R., Sweeney, F.D., Panier, S., Mendez, M., Wildenhain, J., Thomson, T.M., et al. (2007). Orchestration of the DNA-damage response by the RNF8 ubiquitin ligase. Science *318*, 1637–1640.

Koonin, E.V., Altschul, S.F., and Bork, P. (1996). BRCA1 protein products. Functional motifs. Nat. Genet. *13*, 266–268.

Kumagai, A., and Dunphy, W.G. (2000). Claspin, a novel protein required for the activation of Chk1 during a DNA replication checkpoint response in *Xenopus* egg extracts. Mol. Cell *6*, 839–849.

Kumagai, A., Lee, J., Yoo, H.Y., and Dunphy, W.G. (2006). TopBP1 activates the ATR-ATRIP complex. Cell *124*, 943–955.

Leung, C.C., and Glover, J.N. (2011). BRCT domains: easy as one, two, three. Cell Cycle *10*, 2461–2470.

Leung, C.C., Gong, Z., Chen, J., and Glover, J.N. (2011). Molecular basis of BACH1/FANCJ recognition by TopBP1 in DNA replication checkpoint control. J. Biol. Chem. *286*, 4292–4301.

Lou, Z., Chini, C.C., Minter-Dykhouse, K., and Chen, J. (2003). Mediator of DNA damage checkpoint protein 1 regulates BRCA1 localization and phosphorylation in DNA damage checkpoint control. J. Biol. Chem. *278*, 13599–13602.

Mailand, N., Bekker-Jensen, S., Faustrup, H., Melander, F., Bartek, J., Lukas, C., and Lukas, J. (2007). RNF8 ubiquitylates histones at DNA double-strand breaks and promotes assembly of repair proteins. Cell *131*, 887–900.

Manke, I.A., Lowery, D.M., Nguyen, A., and Yaffe, M.B. (2003). BRCT repeats as phosphopeptide-binding modules involved in protein targeting. Science *302*, 636–639.

Mesquita, R.D., Woods, N.T., Seabra-Junior, E.S., and Monteiro, A.N. (2010). Tandem BRCT domains: DNA's praetorian guard. Genes Cancer *1*, 1140–1146.

Müller, S.M., Galliardt, H., Schneider, J., Barisas, B.G., and Seidel, T. (2013). Quantification of Förster resonance energy transfer by monitoring sensitized emission in living plant cells. Front. Plant Sci. *4*, 413.

Na, Z., Pan, S., Uttamchandani, M., and Yao, S.Q. (2014). Discovery of cell-permeable inhibitors that target the BRCT domain of BRCA1 protein by using a small-molecule microarray. Angew. Chem. Int. Ed. *53*, 8421–8426.

Na, Z., Peng, B., Ng, S., Pan, S., Lee, J.S., Shen, H.M., and Yao, S.Q. (2015). A small-molecule protein-protein interaction inhibitor of PARP1 that targets its BRCT domain. Angew. Chem. Int. Ed. *54*, 2515–2519.

Narvaez, A.J., Ber, S., Crooks, A., Emery, A., Hardwick, B., Guarino Almeida, E., Huggins, D.J., Perera, D., Roberts-Thomson, M., Azzarelli, R., et al. (2017). Modulating protein-protein interactions of the mitotic polo-like kinases to target mutant KRAS. Cell Chem. Biol. *24*, 1017–1028.e7.

Nioche, P., Liu, W.Q., Broutin, I., Charbonnier, F., Latreille, M.T., Vidal, M., Roques, B., Garbay, C., and Ducruix, A. (2002). Crystal structures of the SH2 domain of Grb2: highlight on the binding of a new high-affinity inhibitor. J. Mol. Biol. *315*, 1167–1177.

Paster, W., Paar, C., Eckerstorfer, P., Jakober, A., Drbal, K., Schütz, G.J., Sonnleitner, A., and Stockinger, H. (2009). Genetically encoded Förster resonance energy transfer sensors for the conformation of the Src family kinase Lck. J. Immunol. *182*, 2160–2167.

Peng, M., Litman, R., Jin, Z., Fong, G., and Cantor, S.B. (2006). BACH1 is a DNA repair protein supporting BRCA1 damage response. Oncogene *25*, 2245–2253.

Polato, F., Callen, E., Wong, N., Faryabi, R., Bunting, S., Chen, H.T., Kozak, M., Kruhlak, M.J., Reczek, C.R., Lee, W.H., et al. (2014). CtIP-mediated resection is essential for viability and can operate independently of BRCA1. J. Exp. Med. *211*, 1027–1036.

Rappas, M., Oliver, A.W., and Pearl, L.H. (2011). Structure and function of the Rad9-binding region of the DNA-damage checkpoint adaptor TopBP1. Nucleic Acids Res. *39*, 313–324.

Reczek, C.R., Szabolcs, M., Stark, J.M., Ludwig, T., and Baer, R. (2013). The interaction between CtIP and BRCA1 is not essential for resection-mediated DNA repair or tumor suppression. J. Cell Biol. *201*, 693–707.

Rodriguez, M., Yu, X., Chen, J., and Songyang, Z. (2003). Phosphopeptide binding specificities of BRCA1 COOH-terminal (BRCT) domains. J. Biol. Chem. *278*, 52914–52918.

San Filippo, J., Sung, P., and Klein, H. (2008). Mechanism of eukaryotic homologous recombination. Annu. Rev. Biochem. *77*, 229–257.

Sato, K., Sundaramoorthy, E., Rajendra, E., Hattori, H., Jeyasekharan, A.D., Ayoub, N., Schiess, R., Aebersold, R., Nishikawa, H., Sedukhina, A.S., et al. (2012). A DNA-damage selective role for BRCA1 E3 ligase in claspin ubiquitylation, CHK1 activation, and DNA repair. Curr. Biol. *22*, 1659–1666.

Scully, R., Chen, J., Plug, A., Xiao, Y., Weaver, D., Feunteun, J., Ashley, T., and Livingston, D.M. (1997). Association of BRCA1 with Rad51 in mitotic and meiotic cells. Cell *88*, 265–275.

Seidel, S.A., Dijkman, P.M., Lea, W.A., van den Bogaart, G., Jerabek-Willemsen, M., Lazic, A., Joseph, J.S., Srinivasan, P., Baaske, P., Simeonov, A., et al. (2013). Microscale thermophoresis quantifies biomolecular interactions under previously challenging conditions. Methods *59*, 301–315.

Seitz, S., Waβmuth, P., Plaschke, J., Schackert, H.K., Karsten, U., Santibanez-Koref, M.F., Schlag, P.M., and Scherneck, S. (2003). Identification of microsatellite instability and mismatch repair gene mutations in breast cancer cell lines. Genes Chromosomes Cancer *37*, 29–35.

Shao, Z., Li, F., Sy, S.M., Yan, W., Zhang, Z., Gong, D., Wen, B., Huen, M.S., Gong, Q., Wu, J., et al. (2012). Specific recognition of phosphorylated tail of H2AX by the tandem BRCT domains of MCPH1 revealed by complex structure. J. Struct. Biol. *177*, 459–468.

Shiozaki, E.N., Gu, L., Yan, N., and Shi, Y. (2004). Structure of the BRCT repeats of BRCA1 bound to a BACH1 phosphopeptide: implications for signaling. Mol. Cell *14*, 405–412.

Singh, N., Basnet, H., Wiltshire, T.D., Mohammad, D.H., Thompson, J.R., Heroux, A., Botuyan, M.V., Yaffe, M.B., Couch, F.J., Rosenfeld, M.G., et al. (2012). Dual recognition of phosphoserine and phosphotyrosine in histone variant H2A.X by DNA damage response protein MCPH1. Proc. Natl. Acad. Sci. USA *109*, 14381–14386.

Stewart, G.S., Wang, B., Bignell, C.R., Taylor, A.M., and Elledge, S.J. (2003). MDC1 is a mediator of the mammalian DNA damage checkpoint. Nature *421*, 961–966.

Stucki, M., Clapperton, J.A., Mohammad, D., Yaffe, M.B., Smerdon, S.J., and Jackson, S.P. (2005). MDC1 directly binds phosphorylated histone H2AX to regulate cellular responses to DNA double-strand breaks. Cell *123*, 1213–1226.

Symington, L.S., and Gautier, J. (2011). Double-strand break end resection and repair pathway choice. Annu. Rev. Genet. *45*, 247–271.

Tenzer, A., and Pruschy, M. (2003). Potentiation of DNA-damage-induced cytotoxicity by G2 checkpoint abrogators. Curr. Med. Chem. Anticancer Agents *3*, 35–46.

Venkitaraman, A.R. (2014). Cancer suppression by the chromosome custodians, BRCA1 and BRCA2. Science *343*, 1470–1475.

Wang, B., Matsuoka, S., Ballif, B.A., Zhang, D., Smogorzewska, A., Gygi, S.P., and Elledge, S.J. (2007). Abraxas and RAP80 form a BRCA1 protein complex required for the DNA damage response. Science *316*, 1194–1198.

Williams, R.S., Green, R., and Glover, J.N. (2001). Crystal structure of the BRCT repeat region from the breast cancer-associated protein BRCA1. Nat. Struct. Biol. *8*, 838–842.

Williams, R.S., Lee, M.S., Hau, D.D., and Glover, J.N. (2004). Structural basis of phosphopeptide recognition by the BRCT domain of BRCA1. Nat. Struct. Mol. Biol. *11*, 519–525.

Woods, N.T., Mesquita, R.D., Sweet, M., Carvalho, M.A., Li, X., Liu, Y., Nguyen, H., Thomas, C.E., Iversen, E.S., Jr., Marsillac, S., et al. (2012). Charting the landscape of tandem BRCT domain-mediated protein interactions. Sci. Signal. *5*, rs6.

Wu, Q., Paul, A., Su, D., Mehmood, S., Foo, T.K., Ochi, T., Bunting, E.L., Xia, B., Robinson, C.V., Wang, B., et al. (2016). Structure of BRCA1-BRCT/abraxas complex reveals phosphorylation-dependent BRCT dimerization at DNA damage sites. Mol. Cell *61*, 434–448.

Yarden, R.I., Pardo-Reoyo, S., Sgagias, M., Cowan, K.H., and Brody, L.C. (2002). BRCA1 regulates the G2/M checkpoint by activating Chk1 kinase upon DNA damage. Nat. Genet. *30*, 285–289.

You, Z., Shi, L.Z., Zhu, Q., Wu, P., Zhang, Y.W., Basilio, A., Tonnu, N., Verma, I.M., Berns, M.W., and Hunter, T. (2009). CtIP links DNA double-strand break sensing to resection. Mol. Cell *36*, 954–969.

Yu, X., and Chen, J. (2004). DNA damage-induced cell cycle checkpoint control requires CtIP, a phosphorylation-dependent binding partner of BRCA1 C-terminal domains. Mol. Cell. Biol. *24*, 9478–9486.

Yu, X., Chini, C.C., He, M., Mer, G., and Chen, J. (2003). The BRCT domain is a phospho-protein binding domain. Science *302*, 639–642.

Yu, X., Wu, L.C., Bowcock, A.M., Aronheim, A., and Baer, R. (1998). The C-terminal (BRCT) domains of BRCA1 interact in vivo with CtIP, a protein implicated in the CtBP pathway of transcriptional repression. J. Biol. Chem. *273*, 25388–25392.

Yuan, Z., Kumar, E.A., Campbell, S.J., Palermo, N.Y., Kizhake, S., Mark Glover, J.N., and Natarajan, A. (2011). Exploiting the P-1 pocket of BRCT domains toward a structure guided inhibitor design. ACS Med. Chem. Lett. *2*, 764–767.

Zou, L., and Elledge, S.J. (2003). Sensing DNA damage through ATRIP recognition of RPA-ssDNA complexes. Science *300*, 1542–1548.

Zou, Y., Shao, Z., Peng, J., Li, F., Gong, D., Wang, C., Zuo, X., Zhang, Z., Wu, J., Shi, Y., et al. (2014). Crystal structure of triple-BRCT-domain of ECT2 and insights into the binding characteristics to CYK-4. FEBS Lett. *588*, 2911–2920.

STAR★METHODS

KEY RESOURCES TABLE

REAGENT or RESOURCE	SOURCE	IDENTIFIER
Antibodies		
Anti-BRCA1 (D9)	Santa Cruz Biotech	Catalog No: sc-6954, RRID: AB_626761
Anti-BRCA1 (OP92)	EMD Millipore	Catalog No: OP92, RRID: AB_10682944
Anti-Rad51	Novus Biologicals	Catalog No: NB100-148, RRID: AB_10002131
Anti-MDC1	Bethyl Labs	Catalog No: A300-051A, RRID: AB_203282
Anti-RPA32/RPA2	Abcam	Catalog No: ab2175, RRID: AB_302873
Goat-anti-mouse Alexa 488	Invitrogen	Catalog No: A11001, RRID: AB_2534069
Goat-anti-rabbit Alexa 488	Invitrogen	Catalog No: A11034, RRID: AB_2576217
Bacterial and Virus Strains		
E. coli BL21(DE3)	NEB	Catalog No: C2527I
E. coli BL21(DE3*)	Thermo Fisher	Catalog No: C6010-03
E. coli C41(DE3)	Lucigen	Catalog No: 60442-1
Chemicals, Peptides, and Recombinant Proteins		
Bractoppin	This study	N/A
CCBT2047	This study	N/A
TAMRA_GGSRSTpSPTFNK-NH2	Designer Bioscience	N/A
GGSRSTpSPTFNK-NH2	Designer Bioscience	N/A
Ac-pSPVF-CONH2	Sigma	N/A
Ac-pSPVF-COOH	Sigma	N/A
Ac-pSPTF-COOH	Sigma	N/A
pSPVF-COOH	Sigma	N/A
ESIYFpTPELYDPEDT-NH2	Designer Bioscience	N/A
PSpYVNVQN-NH2	Designer Bioscience	N/A
SILSDIpSFDKTDEpSLDWDSSLE-NH2	Designer BioScience	N/A
KKATQpSQEY	Designer Bioscience	N/A
TKSVAEpTPVHK	Designer Bioscience	N/A
BRCA1 tBRCT (residues 1646-1859)	This study	N/A
GRB2 SH2 (residues 55-152)	This study	N/A
ECT2 BRCT 0/1/2 (residues 22-326)	This study	N/A
MCPH1 tBRCT 2/3 (residues 640-835)	This study	N/A
TOPBP1 BRCT1/2 (residues 1-290)	This study	N/A
TOPBP1 tBRCT 7/8 (residues 1264–1493)	This study	N/A
pET28a Vector	EMD Biosciences	Catalog No: 69864-3
pGEX-4T-3	GE Healthcare	Catalog No: 28-9545-52
LB	HI media	Catalog No: M575
Kanamycin sulfate	Amresco	Catalog No: 0408
Chloramphenicol	Amresco	Catalog No: 0230
Tris - HCl	Fischer Scientific	Catalog No: 15965
EDTA	Fischer Scientific	Catalog No: 12635
IPTG	Sigma	Catalog No: 15002
Imidazole	Merck	Catalog No: 104716
PMSF	Amresco	Catalog No: 97064-898-EA
DTT	Sigma	Catalog No: 43815

(*Continued on next page*)

Continued

REAGENT or RESOURCE	SOURCE	IDENTIFIER
Lysozyme	Sigma	Catalog No: L6876
Protease inhibitor cocktail tablet	Roche	Catalog No: 11697498001
Sodium chloride	Hi Media	Catalog No: MB023-1
Sodium di-hydrogen phosphate	Hi Media	Catalog No: TC068-1KG
SDS Page Precast gels	Biorad	Catalog No: 4561033
DMSO	Merck	Catalog No: 102952
Tween-20	Sigma	Catalog No: P2287
Glycerol	Fischer Scientific	Catalog No: 11005
3 color pre-stained protein ladder	Puregene	Catalog No: PG-PMT2962
Calcein AM	Thermo Fisher Scientific	Catalog No: C3100MP
DAPI	Sigma	Catalog No: D9542
Thymidine	Acros Organics	Code: 226740250
Doxycycline hyclate	Sigma	Catalog No: D9891
Mowiol 40-88	Sigma	Catalog No: 324590
Hygromycin B	Thermo Fisher Scientific	Catalog No: 10687010
Blasticidin	InvivoGen	Catalog Code: ant-bl
Zeocin	Thermo Fisher Scientific	Catalog No: R25001
Critical Commercial Assays		
MICROPLATE, 384 WELL, PP, F-BOTTOM, BLACK,	Greiner Bio-One	Catalog No: 781209
Anti-6xHis Alpha LISA Acceptor beads	PerkinElmer	Part No: AL128C
AlphaScreen Streptavidin Donor beads	PerkinElmer	Part No: 6760002S
96 well plates, half-area white plates	Corning	Catalog No: CLS3642
Monolith NT Protein Labeling Kit RED- NHS	Nanotemper	Catalog No: L001
Monolith NT.115 MST Premium Coated Capillaries	Nanotemper	Catalog No: MO-K005
Experimental Models: Cell Lines		
HEK Parental (Flp-In™ T-REx™ 293)	Thermo Fisher Scientific	Catalog No: R78007
CAL-51 cells	Leibniz Institute DSMZ-German Collection of Microorganisms and Cell Cultures, GmbH	DSMZ No: ACC302 (DSMZ)
Recombinant DNA		
pcDNA5/FRT/TO	Invitrogen	Catalog No: V6520-20
pOG44	Invitrogen	Catalog No: V6005-20
pcDNA5/FRT/TO-mCherry-BRCT(tBRCA1)	GeneArt	N/A
pcDNA5/FRT/TO- mCherry-BRCT(tMDC1)	GeneArt	N/A
pReceiver-C-HaloTag-BRCA1 [NM_007294.3]	GeneCopoeia	Catalog No: EX-H0047-M50
Software and Algorithms		
Schrödinger small molecule drug discovery suite (Schrödinger Release 2015-3]	Schrödinger	https://www.schrodinger.com/suites/small-molecule-drug-discovery-suite
Pymol Ver:1.7.4	Schrödinger	https://www.schrodinger.com/suites/pymol
HCS Studio 2.0	Thermo Fisher Scientific	Catalog No: SX000041A
Other		
96 well imaging plates	Eppendorf	Catalog No: 0030741030

CONTACT FOR REAGENT AND RESOURCE SHARING

Further information and requests for resources and reagents should be directed to and will be fulfilled by the Lead Contact, Ashok R. Venkitaraman (ashokv@ncbs.res.in).

EXPERIMENTAL MODEL AND SUBJECT DETAILS

Compounds

Compounds were synthesized by O2h Discovery (Ahmedabad, India), and validated by liquid chromatography coupled to mass spectrometry (LC/MS) and 1H-NMR. Synthetic methods are provided below under Method Details. All compounds were >95% pure as determined by high-performance liquid chromatography (HPLC). Stock solutions were prepared from dry powder in 100% DMSO at 50mM concentration. For primary screens by FP, an initial stock solution of 5mM compound in 100% DMSO was diluted to 125uM in 2% DMSO. For Alpha assays, 5x concentrations of each of the half-logarithmic dilutions of the compound in 100% DMSO were diluted to the indicated assay concentrations in 2% DMSO. For MST assays, stock solutions were diluted to the indicated assay concentrations in 2% DMSO. For cell-based experiments, 20mM stocks of Bractoppin and CCBT2047 in 100% DMSO were diluted in growth media (DMEM supplemented with 10%FBS, 2mM Glutamine) to the indicated concentrations (0.5% DMSO final concentration), thoroughly mixed, and spun at 13,000rpm for 10 seconds before use.

Antibodies

Antibodies used for immunofluorescence analysis at the indicated dilutions were: BRCA1 (sc-6954, Santa Cruz Biotech, 1:600), BRCA1 (OP92, EMD Millipore, 1:200), MDC1 (A300-051A, Bethyl Labs, 1:250), RPA32/RPA2 (ab2175, Abcam, 1:250), Rad51 (NB100-148, Novus Biologicals, 1:1000), Histone H3 (Ser10] (ab5176, Abcam, 1:200). For the Western blotting analysis of protein expression in stable clones, mCherry antibody (GTX128509, GeneTex) was used at 1:2000.

Expression Constructs

Tetracycline (Tet)-inducible plasmids encoding mCherry fused to either wild-type of mutant BRCA1 or MDC1 tBRCT domains were prepared in the pcDNA5/FRT/TO-mCherry vector by gene synthesis (GeneArt, Regensburg, Germany). Briefly, synthetic polynucleotides encoding SV40-NLS (3X)-BRCA1 tBRCT (aa 1620-1862) or SV40-NLS (3X) MDC1 tBRCT (aa 1875-2089) were cloned between the *BamH*1 and *Xho*I restriction sites of the vector. The unimolecular FRET biosensor was also synthesized. Briefly, Tag-BFP was attached N-terminally to a BACH1 (aa 985-1000) peptide containing the Ser990 phosphosite, and C-terminally to 3x SV40-NLS, followed by the BRCA1 tBRCT domain (aa 1643-1862) and Tag-GFP2 (Figure 4A).

Cell Lines and Cell Culture

The Flp-In™ T-REx™ 293 Cell Line was procured from Thermo Fisher Scientific (R78007) and maintained in DMEM supplemented with 10%FBS, 2mM L-glutamine, Blasticidin (15μg/ml) and Zeocin (100μg/ml) at 37°C with 5% CO_2. Stable cell lines were generated by co-transfecting pOG44 with pCDNA5/FRT/TO vectors encoding the gene of interest (see Expression constructs) in a 9:1 ratio using FuGENE HD transfection reagent (E2311, Promega). After 10 days of selection using Blasticidin (15μg/ml) and Hygromycin B (50μg/ml), viable colonies were expanded, and assayed for the loss of β-galactosidase activity and Zeocin resistance to identify clones with stable integration of plasmid. Protein expression was induced with Doxycycline (1μg/ml for 48 h). CAL-51 cells (maintained in DMEM supplemented with 10%FBS, 2mM L-glutamine at 37°C with 5% CO_2) were transfected with a construct encoding wild-type full-length human BRCA1, and a single-cell clone (CAL-51 clone 60) isolated by neomycin selection was used for further studies. Both the Flp-In™ T-REx™ 293 and CAL-51 clone 60 cells were authenticated at the DNA Forensics Laboratory Pvt. Ltd., New Delhi, India using short tandem repeat (STR) profiling at the 8 core loci plus amelogenin specified by Capes-Davies et al. (2013). Flp-In™ T-REx™ 293 cells were confirmed to be female, and were an exact match (15/15 alleles) for the HEK-293 (CRL-1573) human cell line in the reference database. CAL-51 clone 60 cells were also confirmed to be female, and were an 80% match (12/15 alleles) with the CAL-51 human cell line in the reference database confirming relatedness (Capes-Davies et al., 2013), and consistent with mutational drift and microsatellite instability observed in CAL-51 cells during passage in culture (Seitz et al., 2003; Gorringe et al., 2005).

Cell Irradiation

Cells were irradiated to the indicated doses using either the Blood Irradiator 2000 with Cobalt-60 (Board of Radiation and Isotope Technology, Department of Atomic Energy, Government of India) at an effective dose rate of 3.9Gy/min, or with an X-ray generator (Xstrahl, RS225) at a dose rate of 1.5Gy/min.

METHOD DETAILS

Where applicable, information concerning replication of experiments, the sample size analysed, and the statistical method used for comparisons is provided in the figure legends.

Chemical Synthesis

Detailed methods for compound synthesis are as follows.

Synthesis of Bractoppin: (4- (2-fluorobenzyl)piperazin-1-yl) (2-phenyl-1H-benzo[d]imidazol-6-yl) methanone

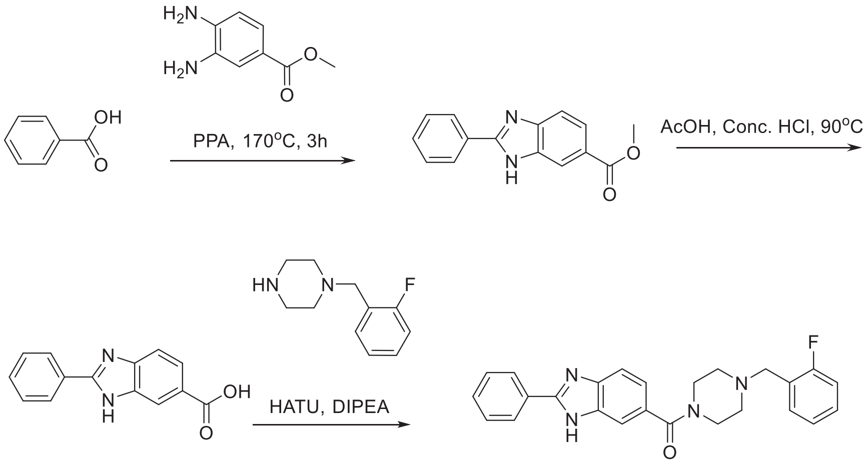

CCBT2008

Synthesis of methyl 2-phenyl-1H-benzo[d]imidazole-6-carboxylate: In a vial the mixture of benzoic acid (0.367 g), methyl 3,4-diaminobezoate (0.5 g) and PPA (2 g) was heated to 170°C for 3 h, TLC (MDC : MeOH = 9:1) indicated that starting material was consumed. The reaction mixture was poured into saturated $NaHCO_3$ solution followed by extraction with ethyl acetate (20mL x 3). The combined organic phase was washed with brine (30mL x 2), dried with anhydrous Na_2SO_4, filtered and concentrated in vacuum to afford methyl 2-phenyl-1H-benzo[d]imidazole-6-carboxylate (0.12 g, crude) obtained as an off-white solid.

Synthesis of 2-phenyl-1H-benzo[d]imidazole-6-carboxylic acid: The mixture of methyl 2-phenyl-1H-benzo[d]imidazole-6-carboxylate (0.1 g), concentrated HCl (7 mL), acetic acid (6 mL) was heated to 90°C for 3 h, TLC (MDC:MeOH = 9:1) indicated that starting material was consumed. The reaction mixture was neutralized by saturated $NaHCO_3$ solution followed by extraction with ethyl acetate (20 mL x 3). The combined organic phase was washed with brine (30 mL x 3), dried with anhydrous Na_2SO_4, filtered and concentrated in vacuum to afford methyl 2-phenyl-1H-benzo[d]imidazole-6-carboxylatic acid (0.07 g, crude) obtained as an off-white solid.

Synthesis of (4-(2-fluorobenzyl) piperazin-1-yl) (2-phenyl-1H-benzo[d] imidazol-6-yl) methanone: To a solution of methyl 2-phenyl-1H-benzo[d]imidazole-6-carboxylic acid (0.07 g) in DMF was added 1- (2-fluorobenzyl) piperazine (0.06 g) and HATU (0.17 g). Reaction mixture was cooled to 0°-5°C followed by the addition of DIPEA (0.1 mL) and stirred at same temperature for 2 h, TLC (MDC: MeOH = 9:1) indicated that both starting materials were consumed. Reaction mixture was poured into water followed by extraction with ethyl acetate (10 mL x 3). The combined organic phase was washed with brine (40 mL x 2), dried with anhydrous Na_2SO_4, filtered and concentrated in vacuum to afford crude which was purified by flash chromatography where the product eluted at 3% MeOH in MDC to afford of (4- (2-fluorobenzyl)piperazin-1-yl) (2-phenyl-1H-benzo[d] imidazol-6-yl) methanone (0.035 g). LCMS: $(M+H^+)$: 415.3. 1H NMR: DMSO-d_6 400 MHz δ: 13.132 (s, 1H), 8.198-8.180 (d, J = 7.2 Hz, 2H), 7.708-7.667 (m, 1H), 7.586-7.726 (m, 4H), 7.446-7.412 (t, J = 6.8 Hz, 1H), 7.341-7.324 (d, J = 6.8 Hz, 1H), 3.581 (s, 3H), 2.442 (s,1H), 1.225 (s,1H), HPLC Purity; 100%

Synthesis of CCBT2009: (4- (2-fluorobenzyl)piperazin-1-yl) (2-isopropyl-1H-benzo[d]imidazol-6-yl) methanone

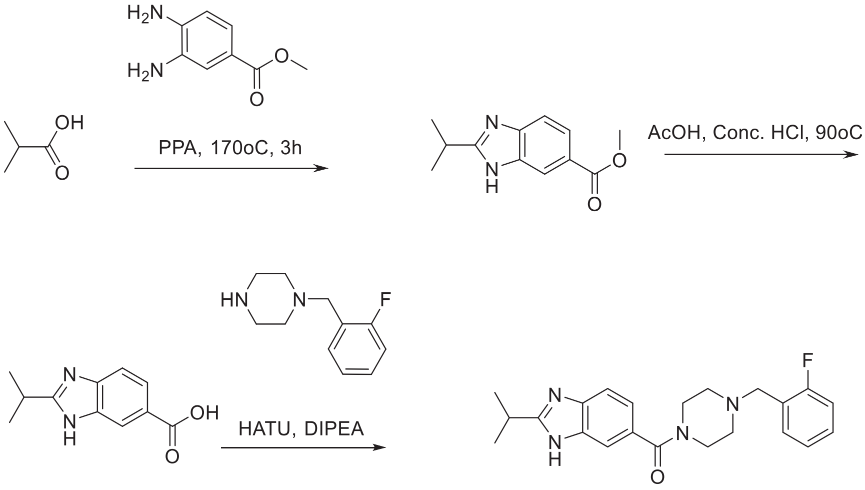

CCBT2009

Synthesis of methyl 2-isopropyl-1H-benzo[d]imidazole-6-carboxylate: In a vial the mixture of isobutyric acid (0.15 g), methyl 3,4-diaminobezoate (0.3 g) and PPA (2 g) was heated to 170°C for 3 h, TLC (MDC:MeOH = 9:1) indicated that starting material was consumed. Reaction mixture was poured into saturated $NaHCO_3$ solution followed by extraction with ethyl acetate (20 mL x 4). The combined organic phase was washed with brine (10 mL x 2), dried with anhydrous Na_2SO_4, filtered and concentrated in vacuum to afford methyl 2-isopropyl-1H-benzo[d]imidazole-6-carboxylate (0.45 g, crude) obtained as an off-white solid.

Synthesis of methyl 2-isopropyl-1H-benzo[d]imidazole-6-carboxylic acid: The mixture of methyl 2-isopropyl-1H-benzo[d]imidazole-6-carboxylate (0.45 g), concentrated HCl (7 mL), acetic acid (6 mL) was heated to 90°C for 3 h, TLC (MDC : MeOH = 9:1) indicated that starting material was consumed. Reaction mixture was neutralized by saturated $NaHCO_3$ solution (pH~7) followed by extraction with ethyl acetate (20 mL x 4). The combined organic phase was washed with brine (10 mL x 3), dried with anhydrous Na_2SO_4, filtered and concentrated in vacuum to afford methyl 2-isopropyl-1H-benzo[d]imidazole-6-carboxylic acid (0.24 g, crude) obtained as an off-white solid.

Synthesis of (4- (2-fluorobenzyl)piperazin-1-yl) (2-isopropyl-1H-benzo[d]imidazol-6-yl) methanone: To a solution of 2-isopropyl-1H-benzo[d]imidazole-6-carboxylic acid (0.24 g) in DMF (5 mL) was added 1- (2-fluorobenzyl) piperazine (0.228 g), HATU (0.67 g). Reaction mixture was cooled to 0°C-5°C followed by addition of DIPEA (0.4 mL) and stirred at same temperature for 2 h, TLC (MDC: MeOH = 9:1) indicated that both starting materials were consumed. Reaction mixture was poured into water followed by extraction with ethyl acetate (10 mL x 3). The combined organic phase was washed with brine (100 mL x 2), dried with anhydrous Na_2SO_4, filtered and concentrated in vacuum to afford CCBT2009 (0.015g) obtained as white solid after purification by preparative HPLC. LCMS: $(M+H^+)$: 381.40. 1H NMR: DMSO-d_6 400 MHz δ: 12.434 (s, NH), 7.516-7.495 (d, *J* = 8.4 Hz, 2H), 7.465-7.429 (t, *J* = 7.2 Hz, 1H) 7.363-7.349 (d, *J* = 5.6 Hz, 1H), 6.556 (s, 1H), 3.214-3.110 (m,1H), 2.544 (s, 2H), 1.355-1.338 (d, *J* = 6.8 Hz, 6H). HPLC Purity; 99.54%

Synthesis of CCBT2029: (4- (2-fluorobenzyl)piperazin-1-yl) (1H-indol-6-yl)methanone

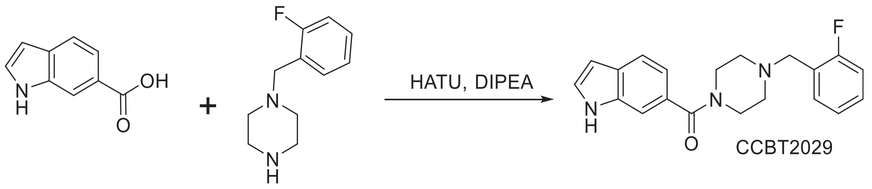

To a solution of indole 5-carboxylic acid (0.5 g, 3.0 mmol) in DMF (10 mL) was added HATU (2.35 g, 6.1 mmol), DIPEA (1.19 g, 9.2 mmol) at 0°C, and further added 1- (2-fluorobenzyl)piperazine (0.72 g, 3.7 mmol) and stirred at room temperature for 1 h, TLC (Chloroform: Methanol = 9:1) indicated the starting material was consumed. The reaction mixture was poured into cold water and extracted with ethyl acetate. The combined organic phase was washed with brine (100 mL x 2), dried with anhydrous Na_2SO_4, filtered and concentrated in vacuum to afford the crude which was purified by flash chromatography where the product eluted at 65% ethyl acetate in hexane to give (4- (2-fluorobenzyl)piperazin-1-yl) (1H-indol-6-yl)methanone (0.3 g) obtained as light yellow oil. HPLC Purity; 99.39%

Synthesis of CCBT2047: (4-isobutylpiperazin-1-yl)(2-phenyl-1H-benzo[d]imidazol-5-yl) methanone

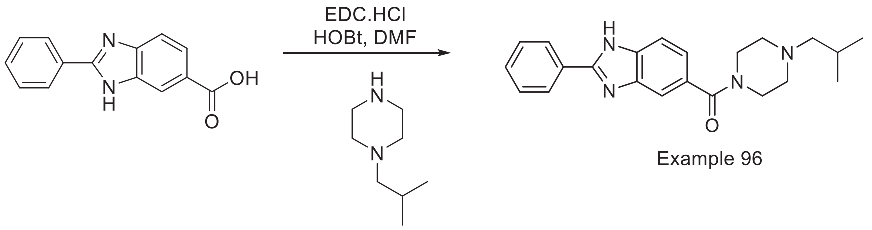

To a solution of 2-phenyl-1H-benzo[d]imidazole-6-carboxylic acid (0.1 g, 0.42 mmol, 1.0 eq) in DMF (5 mL) was added EDC.HCl (0.08 g, 0.46 mmol, 1.1 eq) and HoBt (0.02 g, 0.21 mmol, 0.5 eq) and stirred at room temperature for 30 min. To this N-isobutylpiperazine (0.06 g, 0.42 mmol, 1.0 eq) and DIPEA (0.2 mL, 1.26 mmol, 3.0 eq) was charged. The mixture was stirred at room temperature for 18 h, TLC ($CHCl_3$: MeOH = 9:1) indicated the starting material was consumed. Reaction mixture was poured into water followed by extraction with ethyl acetate (30 mL*3). The combined organic phase was washed with brine (40 mL*2), dried with anhydrous Na_2SO_4, filtered and concentrated in vacuum to afford the crude which was purified by flash chromatography where the product eluted at 3.5% MeOH in chloroform followed by trituration with n-pentane to afford the product (0.022g) as light brown solid. LCMS: $(M+H^+)$: 363.4. ^1H NMR: DMSO-d_6 400 MHz δ: 13.127 (s, 1H), 8.202-8.183 (d, J= 7.6Hz, 2H), 7.713-7.673 (t, J= 8.0Hz, 2H), 7.592-7.504 (m, 4H), 7.277-7.211 (q, J= 18Hz, 1H), 3.508 (broad s, 4H), 2.362-2.332 (t, J= 10Hz, 4H), 2.077-2.063 (d, J= 5.6Hz, 2H), 1.793-1.777 (broad d, J= 6.4Hz, 1H), 0.871-0.861 (d, J= 4.0Hz, 6H), HPLC Purity; 98.83%

Synthesis of CCBT2082: (4-(2-fluorobenzyl)piperazin-1-yl)(2-(pyridin-4-yl)-1H-benzo[d]imidazol-5-yl)methanone

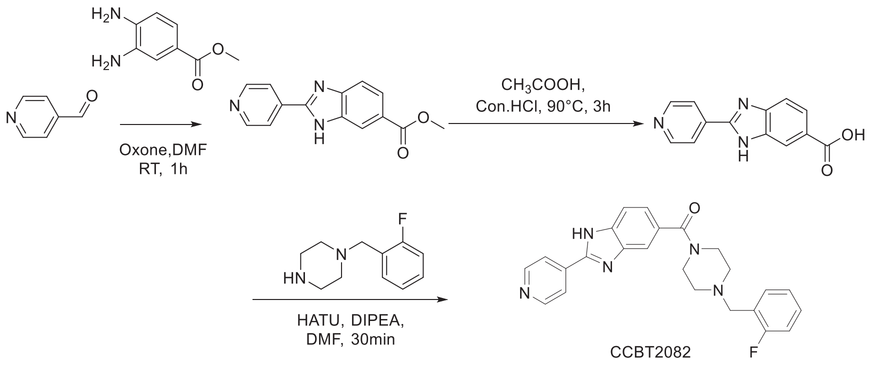

Synthesis of methyl 2-(pyridin-4-yl)-1H-benzo[d]imidazole-6-carboxylate: To a solution of methyl 3,4-Diaminobenzoate (0.284 g, 2.406 mmol, 1.0 eq) in DMF (12 mL) were added Pyridine-4-carboxaldehyde (0.4 g, 2.647 mmol, 1.1 eq) and oxone (0.962 g, 1.564 mmol, 0.65 eq) at room temperature. The mixture was stirred at room temperature for 2 h, TLC (Hexane: Ethylacetate = 5:5) indicated the starting material was consumed. The mixture was poured on to the saturated $NaHCO_3$ solution (20 mL) followed by extraction with ethyl acetate (100 mL*2). The combined organic phase was washed with brine (30 mL*2), dried with anhydrous Na_2SO_4, filtered and concentrated in vacuum to afford the product (0.5g, crude) as light yellow solid.

Synthesis of 2-(pyridin-4-yl)-1H-benzo[d]imidazole-6-carboxylic acid:A solution of methyl 2-(pyridin-4-yl)-1H-benzo[d]imidazole-6-carboxylate (0.3 g) in concentrated HCl (5 mL) and acetic acid (5 mL) was heated at 90°C for 3 h, TLC (Dichloromethane: Methanol= 9:1) indicated that starting material was consumed. The reaction mixture was concentrated under vacuum and the traces acetic acid was further removed by azeotropic distillation with dichloromethane (10 mL). The obtained solid was dried under vacuum to afford the product (0.280 g, crude) as brown solid

Synthesis of (4-(2-fluorobenzyl)piperazin-1-yl)(2-(pyridin-4-yl)-1H-benzo[d]imidazol-5-yl) methanone: To a solution of 2-(pyridin-4-yl)-1H-benzo[d]imidazole-6-carboxylic acid (0.190 g, 0.771 mmol, 1.5 eq) in DMF (5 mL) were added HATU (0.590 g, 1.543 mmol, 3.0 eq) and DIPEA (0.400 g, 3.087 mmol, 6.0 eq) at 0°C. The reaction mixture was stirred at 0°C for 30 min. 1-(2-fluorobenzyl)piperazine (0.1 g, 0.514 mmol, 1.0 eq) was added in to the reaction mixture and stirred at room temperature for 2 h, TLC (Dichloromethane: Methanol= 9:1) indicated that both starting materials were consumed. The reaction mixture was poured in to the saturated $NaHCO_3$ solution (30 mL) and product was extracted by ethyl acetate (50 mL*3). The combined organic phase was washed with brine (100 mL*2), dried with anhydrous Na2SO4, filtered and concentrated in vacuum to afford crude. This crude material was purified by flash chromatography where the product eluted at 7% methanol in chloroform. The pure product fraction was concentrated under vacuum to afford the product (0.04 g, pure) as off white solid. LCMS: ($M+H^+$): 416.3. ^1H NMR: DMSO-d_6 400 MHz δ: 13.512 (s, 1H), 8.786 (d, J=5.2 Hz, 2H), 8.117 (d, J=5.2 Hz, 2H), 7.592-7.786 (m, 2H), 7.433 (t, J = 7.2 Hz, 1H), 7.159-7.380 (m, 4H), 3.410-3.810 (m, 6H), 2.350-2.510 (m, 4H), HPLC Purity; 97.65%

Synthesis of CCBT2084: (4-(2-fluorobenzyl)piperazin-1-yl)(2-morpholino-1H-benzo[d]imidazol-5-yl) methanone

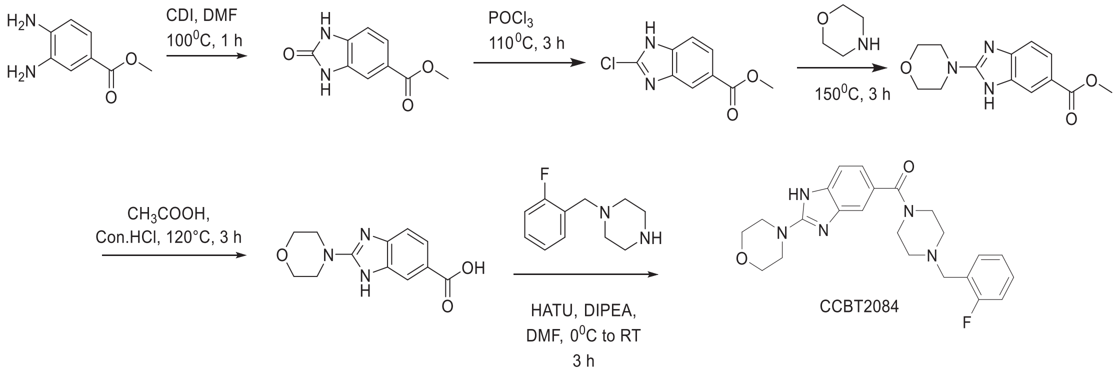

Synthesis of methyl 2-oxo-2,3-dihydro-1H-benzo[d]imidazole-5-carboxylate: To a solution of Methyl-3,4-diaminobenzoate (1.5 g, 9.02 mmol, 1.0 eq) in DMF (5 mL) was added CDI (2.2 g, 13.54 mmol, 1.5 eq) at room temperature. The reaction mixture was heated at 100°C for 1 h, TLC (100% Ethyl acetate) indicated the starting material was consumed. The reaction mixture was poured into ice-cold water and obtained precipitates were collected by filtration, washed with distilled water and dried to afford methyl 2-oxo-2,3-dihydro-1H- benzo[d]imidazole-5-carboxylate (1.52 g, pure).

Synthesis of methyl 2-chloro-1H-benzo[d]imidazole-5-carboxylate: A suspension of methyl 2-oxo-2,3-dihydro-1H-benzo[d]imidazole-5-carboxylate (1.5 g) in $POCl_3$ (15 mL) was heated at 120°C for 3 h, TLC (Hexane: Ethyl acetate= 5:5) indicated the starting

material was consumed. The reaction mixture was concentrated under vacuum and obtained residue was suspended in saturated $NaHCO_3$ solution (50ml). The resulting precipitates were collected by filtration, washed with distilled water and dried to afford methyl 2-chloro-1H-benzo[d]imidazole-5-carboxylate (1.38 g, pure).

Synthesis of methyl 2-morpholino-1H-benzo[d]imidazole-6-carboxylate: Methyl 2-chloro-1H-benzo[d]imidazole-5-carboxylate (0.367 g, 1.0 eq) and morpholine (2 mL) was heated at 150°C for 3 h, TLC (100% Ethyl acetate) indicated that starting material was consumed. The reaction mixture was poured into water followed by extraction with 10% MeOH in MDC (20 mL*3). The combined organic phase was washed with water (30 mL), dried with anhydrous Na_2SO_4, filtered and concentrated in vacuum. The obtained crude material was purified by flash chromatography, where the product was eluted at 80% EtoAc in Hexane. The obtained product fraction was concentrated under vacuum to afford methyl 2-morpholino-1H-benzo[d]imidazole-6-carboxylate (0.1 g, pure) as light yellow liquid.

Synthesis of 2-morpholino-1H-benzo[d]imidazole-6-carboxylic acid: A solution of methyl 2-morpholino-1H-benzo[d]imidazole-6-carboxylate (0.1 g) in concentrated HCl (0.6 mL) and acetic acid (0.6 mL) was heated to 120°C for 3 h, TLC (Dichloromethane: Methanol= 9:1) indicated that starting material was consumed. The reaction mixture was concentrated under vacuum and the traces acetic acid was further removed by azeotropic distillation with dichloromethane (50 ml). The obtained solid was dried under vacuum to afford the product (0.11 g, crude) as light yellow solid.

Synthesis of (4-(2-fluorobenzyl)piperazin-1-yl)(2-morpholino-1H-benzo[d]imidazol-5-yl) methanone: To a solution of 2-morpholino-1H-benzo[d]imidazole-6-carboxylic acid (0.1 g, 0.4 mmol, 1.0 eq) in DMF (5 mL) were added 1-(2-fluorobenzyl)piperazine (0.08 g, 0.4 mmol, 1.0 eq) and HATU (0.155 g, 0.48 mmol, 1.2 eq) at 0°C. The reaction mixture was stirred for 10 min and DIPEA (0.160 g, 1.2 mmol, 3.0 eq) was added at 0°C. The mixture was stirred at room temperature for 3 h, TLC (Dichloromethane: Methanol= 9:1) indicated the starting material was consumed. Reaction mixture was poured into water followed by extraction with ethyl acetate (30 mL*3). The combined organic phase was washed with brine (40 mL*2), dried with anhydrous Na2SO4, filtered and concentrated in vacuum to afford the crude which was purified by prep-HPLC purification using NH4HCO3 as buffer to afford (4-(2-fluorobenzyl)piperazin-1-yl)(2-morpholino-1H-benzo[d]imidazol-5-yl) methanone (0.030 g) as white solid. LCMS: $(M+H^+)$: 424.45. ^1H NMR: DMSO-d_6 400 MHz δ: 11.799 (br, s, 1H), 7.410-7.480 (m, 1H), 7.310-7.390 (m, 1H), 7.165-7.243 (m, 4H), 7.003-7.022 (d, J= 7.2 Hz 1H), 3.718-3.741 (m, 4H), 3.349-3.650 (m, 12 H), 2.381-2.500 (m, 4H), HPLC Purity; 100%

Synthesis of CCBT2106: (4-(4-hydroxybenzyl)piperazin-1-yl)(2-phenyl-1H-benzo[d] imidazole-6-yl)methanone

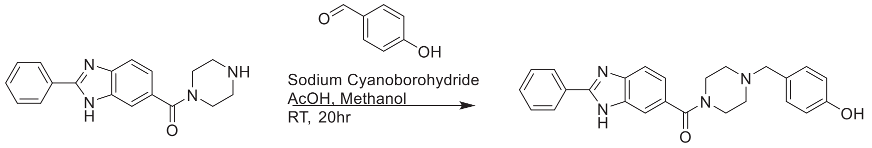

CCBT2106

To a solution of (2-phenyl-1H-benzo[d]imidazol-6-yl)(piperazin-1-yl)methanone (0.3 g, 0.877 mmol, 1.0 eq) and 4-hydroxybenzaldehyde (0.160 g, 1.315 mmol, 1.5 eq) in methanol (5 mL) were added TEA (0.132 g, 1.315 mmol, 1.5 eq) and acetic acid (5 drops) at room temperature. The reaction mixture was stirred at room temperature for 1 h. Sodiumcyanoborohydride (0.273 g, 4.385 mmol, 5.0 eq) was added in to the reaction mixture and stirred for 20 h, TLC (Chloroform: Methanol= 9:1) indicated the starting material was consumed. Reaction mixture was poured into ice cold water and extracted with ethyl acetate (50 mL*3). The combined organic phase was washed with brine (30 mL*2), dried with anhydrous Na_2SO_4, filtered and concentrated in vacuum to afford the crude which was purified by prep-HPLC purification using NH_4HCO_3 as buffer to afford the product (0.90 g) as off white solid. LCMS: $(M+H^+)$: 413.2. ^1H NMR: DMSO-d_6, 400 MHz δ: 13.031 (s, 2H), 8.180-8.231 (m, 4H), 7.504-7.643 (m, 5H), 7.237 (dd, J=1.2 Hz, J=8.4 Hz, 1H), 7.093 (d, J=8.4 Hz, 2H), 6.707 (d, J = 8.4 Hz, 2H), 3.430-3.580 (m, 4H), 2.303-2.445(m, 4H).

Synthesis of CCBT2107: (4-(4-methylbenzyl)piperazin-1-yl)(2-phenyl-1H-benzo[d] imidazol-6-yl)methanone

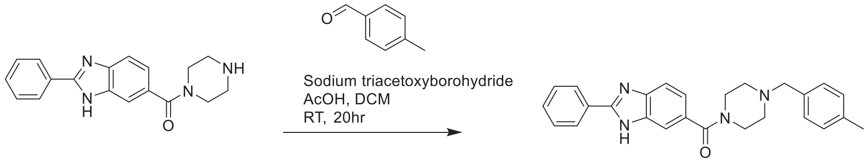

CCBT2107

To a solution of (2-phenyl-1H-benzo[d]imidazol-6-yl)(piperazin-1-yl)methanone (0.25 g, 0.728 mmol, 1.0 eq) and 4-methylbenzaldehyde (0.096 g, 0.801 mmol, 1.1 eq) in DCM (5 mL) were added TEA (0.110 g, 1.092 mmol, 1.5 eq) and acetic acid (5 drops) at room temperature. The reaction mixture was stirred at room temperature for 1 h. Sodium triacetoxyborohydride (0.461 g, 2.184 mmol, 3.0 eq) was added in to the reaction mixture and stirred for 20 h, TLC (Chloroform: Methanol= 9:1) indicated the starting material was consumed. Reaction mixture was poured into ice cold water and extracted with ethyl acetate (50 mL*3). The combined organic phase was washed with brine (30 mL*2), dried with anhydrous Na_2SO_4, filtered and concentrated in vacuum to afford the crude which was purified by prep-HPLC purification using NH_4HCO_3 as buffer to afford the product (0.050 g) as off white solid.

LCMS: $(M+H^+)$: 411.27. ^{1}H NMR: DMSO-d_6, 400 MHz δ: 13.18 (br s, 1H), 8.194 (d, J = 7.2 Hz, 2H), 7.496-7.638 (m, 5H), 7.190-7.241 (m, 3H), 7.120-7.150 (m, 2H), 3.470-3.620 (m, 4H), 3.468(s, 2H), 2.320-2.445 (m, 4H), 2.281 (s, 3H), HPLC Purity; 98.75%

Synthesis of CCBT2905: (R)-(4-(2-fluorobenzyl)-2-isopropylpiperazin-1-yl)(2-methyl-1H-benzo[d]imidazol-5-yl)methanone

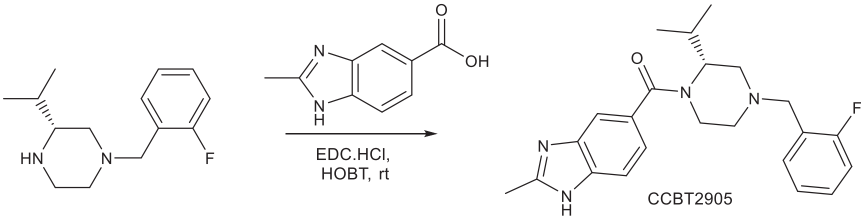

To a stirred solution of 2-Methyl-1H-benzimidazole-5-carboxylic acid (0.6g, 3.4mmol, 1.0 eq) in DMF (20mL) was added EDC.HCl (0.71g, 3.9mmol, 1.1 eq) followed by HOBT (0.23g, 1.7mmol, 0.5 eq) under N_2 gas atmosphere at room temperature and stirred for 30 min. The resulting reaction mixture was added (R)-1-(2-fluorobenzyl)-3-isopropylpiperazine (0.925g, 3.9mmol, 1.15 eq) at room temperature and stirred for 20h. TLC (9:1; chloroform; methanol) indicated the starting material was consumed. The reacting mixture was poured into water and extracted with ethyl acetate (35ml x 3). The organic layers were combined and washed with water, dried over anhydrous sodium sulphate and concentrated under reduced pressure to afford provide crude product, which was purified by preparative HPLC. LCMS: $(M+H^+)$: 395.5. ^{1}H NMR**:** $CDCl_3$ 400 MHz δ: 12.326-12.378 (d, *J* = 20.8 Hz, 1H), 7.438 (br, 2H), 7.306-7.359 (m, 2H), 7.153-7.206 (m, 2H), 7.066-7.118 (m, 1H), 4.218 (br, 1H), 3.503-3.566 (t, *J* = 6 Hz, 3H) , 3.274-3.348 (br, 1H), 2.942 (br, 2H), 2.683 (s, 1H), 2.336-2.405 (br, 1H), 2.179(br, 1H), 1.968-1.998(d, *J* = 12 Hz, 1H), 0.826-0.892 (br, 4H), 0.502-0.648 (br, 2H), HPLC Purity; 96.13%. Note: CCBT2906 was synthesized from (S)-1-(2-fluorobenzyl)-3-isopropylpiperazine and 2-Methyl-1H-benzimidazole-5-carboxylic acid using similar protocol. HPLC Purity; 100%

Synthesis of CCBT2907: (4-(2-fluorobenzyl)piperazin-1-yl)(2-methyl-1H-benzo[d]imidazol-5-yl)methanone

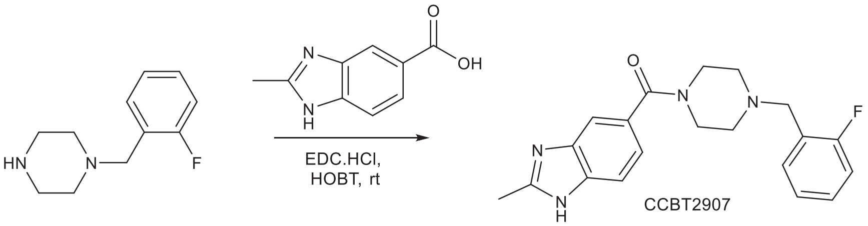

To a stirred solution of 2-Methyl-1H-benzimidazole-5-carboxylic acid (0.5g, 2.8mmol, 1.0 eq) in DMF (10 mL) were added EDC.HCl (0.6 g, 3.11 mmol, 1.1 eq) followed by HOBT (0.19 g, 1.4mmol, 0.5 eq) under N_2 atmosphere at room temperature and stirred for 30 min. The resulting reaction mixture was added 1-(2-fluorobenzyl)piperazine (0.63g, 3.2mmol, 1.15 eq) at room temperature and stirred for 20 h. TLC (9:1; chloroform; methanol) indicated the starting material was consumed. The reacting mixture was poured into water and extracted with ethyl acetate (35 ml x 3). The organic layers were combined and washed with water, dried over anhydrous sodium sulphate and concentrated under reduced pressure to afford crude S2907 which was purified preparative HPLC. LCMS: $(M+H^+)$: 353.5; ^{1}H NMR: 12.382 (d, *J* = 4.8 Hz, 1H), 7.496-7.528 (m, 1H), 7.409-7.44 (m, 2H), 7.306-7.362 (m, 1H), 7.113-7.205 (m, 3H), 3.573 (s, 3H), 3.510 (br, 3H), 2.420 (br, 4H), HPLC Purity; 99.94%

Synthesis of CCBT2908: 5-((4-(2-fluorobenzyl)piperazin-1-yl)methyl)-2-methyl-1H-benzo[d]imidazole

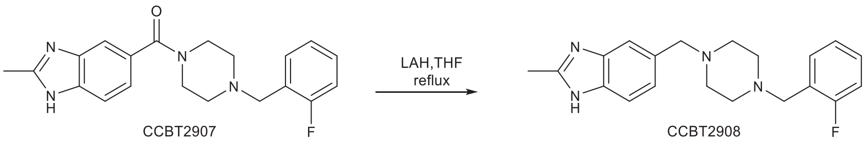

To a stirred solution of CCBT2907 (0.3 g, 0.852 mmol, 1.0 eq) in THF (6mL) was added $LiAlH_4$ (0.85 mL, 2 M in THF, 0.34 mmol, 4.0 eq) at 0°C under N_2 atmosphere, and the resulting reaction mixture was stirred for 3h at room temperature. As observed on TLC, ~50% of CCBT2907 remained unconsumed along with the formation of CCBT2908. Hence, the resulting reaction mixture was further added $LiAlH_4$ (0.85 mL, 2M in THF, 0.34mmol, 4.0 eq) at 0°C, and further heated to 50°C for 5h. TLC (9:1; chloroform; methanol) indicated the starting material was consumed. The resulting reaction mixture was poured in to water and the aqueous layer was extracted with ethyl acetate (25mL x 3). The combined organic layer was then washed with brine, dried over anhydrous sodium sulphate and concentrated under reduced pressure to provide crude which was purified by preparative HPLC. LCMS: $(M+H^+)$: 338.22. ^{1}H NMR: $CDCl_3$ 400 MHz δ: 12.073 (s, 1H), 7.281-7.319 (m, 3H), 7.131-7.184 (m, 2H), 7.018-7.038 (d, *J* = 8 Hz, 1H), 3.512 (s, 4H), 2.457-2.517 (m, 3H), 2.296-2.387 (m, 6H), HPLC Purity; 99.45%

Molecular Docking

Binding mode determination was undertaken using the Glide program from the Schrödinger small molecule drug discovery suite (Schrödinger Release 2015-3). For human BRCA1 tBRCT, unliganded (PDB: 1JNX) and peptide-bound (PDB: 3K0K) forms were superimposed, processed (by adding hydrogen, fixing bond orders, fixing missing atoms and residues, determining and fixing protonation states of side chains) before energetic minimisation of the whole complex using OPLS2005 force field with a maximum permitted rmsd of 0.30 Å. This procedure was carried out using the protein preparation wizard of Maestro 10.3. To dock compounds, the bound peptide and water molecules were deleted. A grid box of size 22 x 27 x 22 $Å^3$ with an inner box (10 x 15 x 10 $Å^3$) centred at X, Y, Z coordinates -21.0, 11.0 and -25.0 was generated that covers the pS-P-T-F residues with default parameters and no constraints. Ligands were drawn and prepared using LigPrep. Ionisation and tautomeric states were carefully selected after inspection. Five conformers were generated for each ligand using Confgen with an OPLS2005 forcefield and a minimum rmsd cutoff of 1.0 Å. Each conformer was then individually docked using the Glide SP protocol, to identify the 10 best poses per ligand. In case of primary screen hits, poses from all the conformers were clustered and each cluster pose was carefully inspected to explain structure activity relationships between analogs.

Molecular Dynamic (MD) Simulations

MD simulations for the complex were carried out using all-atom optimized potential for liquid simulations (OPLS-AA) force field implemented in GPU-accelerated Desmond software (Schrödinger Release 2015-3). Simulations were conducted with a TIP3P explicit solvent model and periodic boundary condition. With 1.2 ps recording intervals, a 5 ns simulation was performed under NPT ensemble with temperature fixed at 300 K and pressure at 1.01 bar. The RESPA integration time step was set at 2 fs and all other parameters including the equilibration step were assigned the default settings available in the Molecular Dynamics wizard of Maestro. Post-simulation analyses were all performed within Desmond software.

Protein Expression and Purification

Synthetic gene constructs encoding the GRB2 SH2 domain (residues 55-152), or the BRCT domains of different human proteins (BRCA1 tBRCT residues 1646-1859, TOPBP1 tBRCT 7/8 residues 1264–1493, TOPBP1 BRCT 1/2 residues 1-290, ECT2 BRCT residues 22-326, MCPH1 tBRCT 2/3 residues 640-835) fused N-terminally to 6x Histidine residues, and codon optimized for expression in *E. coli*, were procured from GeneArt (Regensburg, Germany) in the pET28a expression vector. All constructs were expressed in *E. coli* BL21(DE3) cells grown in LB medium containing 50μg/mL Kanamycin.

BRCA1 tBRCT: 6x His- BRCA1 tBRCT expression was induced in BL21(DE3) strain at 0.6-0.8 OD_{600} with 0.25mM isopropyl β-D-1-thiogalactopyranoside (IPTG) and the culture was grown for 16 h at 18°C. Cells were harvested and the pellet was suspended in ice cold lysis buffer (50mM Tris HCl [pH 7.5], 400mM NaCl, 0.1mM PMSF, 1mM DTT, and 1 protease inhibitor tablet (Roche)). Cells were lysed by sonication on ice and centrifuged at 20,000 rev min^{-1} for 30 min at 4°C to remove cell debris. The supernatant was applied onto a HisTrap HP column (GE Healthcare) pre-equilibrated with a buffer (50mM Tris HCl [pH 7.5], 400mM NaCl, 1mM DTT, and 25mM Imidazole). The column was washed with same buffer until all unbound proteins were removed. The protein of interest was eluted using a linear gradient of up to 100% elution buffer (50mM Tris HCl [pH 7.5], 400mM NaCl, 1mM DTT, and 500mM Imidazole). Protein purity was visualized by running SDS-PAGE. Fractions of sufficient purity were pooled and concentrated to 2 ml using a 10 kDa cutoff Centricon centrifugal filter devices (Millipore). The concentrated protein was further purified using HiLoad 16/600 Superdex-75 prep-grade gel-filtration column (GE Healthcare) pre-equilibrated with 20mM Tris HCl [pH 7.5], 100mM NaCl and 1mM DTT. *TOPBP1 tBRCT 7/8:* A similar procedure was used as for BRCA1 tBRCT, except that: (a) protein expression was induced in BL21(DE3*) strain at 0.6 OD_{600} with 0.2mM IPTG and the culture was grown for 16 h at 18°C, (b) lysis buffer was 50mM Sodium Phosphate [pH-7.5], 300mM NaCl, 0.1mM PMSF, 1mM DTT, 0.1mg/ml Lysozyme, and 1 protease inhibitor tablet, (c) HisTrap column was pre-equilibrated with 50mM Sodium Phosphate [pH-7.5], 300mM NaCl and 20mM Imidazole, and (d) column was eluted with 50mM Sodium Phosphate [pH-7.5], 300mM NaCl and 500mM Imidazole. *TOPBP1 tBRCT 0/1/2:* A similar procedure was used as for BRCA1 tBRCT, except that: (a) protein expression was induced at 1.0 OD_{600} with 0.4mM IPTG and the culture was grown for 16 h at 18°C, (b) lysis buffer was 20mM Sodium Phosphate [pH-7.5], 500mM NaCl, 0.1mM PMSF, 1mM DTT, 0.1mg/ml Lysozyme, and 1 protease inhibitor tablet, (c) HisTrap column was pre-equilibrated with 20mM Sodium Phosphate [pH-7.5], 500mM NaCl and 20mM Imidazole, and (d) column was eluted with 20mM Sodium Phosphate [pH-7.5], 500mM NaCl and 500mM Imidazole. *ECT2 BRCT:* A similar procedure was used as for BRCA1 tBRCT, except that: (a) protein expression was induced in C41(DE3) strain at 1.2 OD_{600} with 1mM IPTG and the culture was grown for 24 h at 14°C, (b) lysis buffer was 50mM HEPES [pH 7.5], 150mM KCl, 100mM PMSF, 10mM Imidazole and 1 protease inhibitor tablet, (c) HisTrap column was pre-equilibrated with 50mM HEPES [pH 7.5], 150mM KCl, 10mM Imidazole and 1mM DTT, and (d) column was eluted with 50mM HEPES [pH 7.5], 150mM KCl, 500mM Imidazole and 1mM DTT, (e) the protein was further purified by anion-exchange chromatography (Mono-Q 10/100 GL, GE Healthcare) with a NaCl gradient (0–1.0 M NaCl in 50 mM HEPES [pH 8.0]), and (f) the concentrated protein was loaded on HiLoad 16/600 Superdex-75 prep-grade gel-filtration column (GE Healthcare) pre-equilibrated with 50mM HEPES [pH 7.5], 150mM KCl and 1mM DTT. *MCPH1 tBRCT 2/3:* A similar procedure was used as for BRCA1 tBRCT, except that: (a) protein expression was induced at 0.6 OD_{600} with 0.25mM IPTG and the culture was grown for 16 h at 18°C, (b) lysis buffer was 20mM Sodium Phosphate [pH-7.5], 500mM NaCl, 0.1mM PMSF, 1mM DTT and 1 protease inhibitor tablet, (c) HisTrap column was pre-equilibrated with 20mM Sodium Phosphate [pH-7.5], 500mM NaCl and 20mM Imidazole, and (d) column was eluted with 20mM Sodium Phosphate [pH-7.5], 500mM

NaCl and 500mM Imidazole. *GRB2 SH2:* A similar procedure was used as for BRCA1 tBRCT, except that: (a) protein expression was induced at 0.6 OD_{600} with 0.25mM IPTG and the culture was grown for 16 h at 18°C, (b) lysis buffer was 50mM Tris HCl [pH 8.0], 500mM NaCl, 100mM PMSF, 5mM beta mercaptoethanol, 10mM Imidazole and 1 protease inhibitor tablet, (c) HisTrap column was pre-equilibrated with 50mM Tris HCl [pH 8.0], 500mM NaCl, 5mM beta mercaptoethanol and 10mM Imidazole, and (d) column was eluted with 50mM Tris HCl [pH 8.0], 150mM NaCl, 5mM beta mercaptoethanol and 500mM Imidazole. Fractions of sufficient purity were pooled and concentrated to 2 ml using a 3 kDa cutoff Centricon centrifugal filter device (Millipore).

Fluorescence Polarization (FP) Assay

FP reactions were conducted in black 384 well plates using the TECAN Freedom EVO 200 dispenser (Tecan). We dispensed 3x working concentrations of BRCA1 tBRCT protein and TAMRA-labeled BACH1 peptide, 10μl each prepared in assay buffer (20mM Tris buffer pH 7.4, 200mM NaCl, 0.05% Tween-20, 2mM DTT) to achieve final concentrations of 75nM and 10nM, respectively. 10 μl of compound at a final concentration of 125μM was added to the plate and incubated for 20 min at room temperature. Relative fluorescence was measured using the TECAN infinite M1000 Pro microplate reader using an excitation wavelength 530 nm, and an emission wavelength of 610 nm. The degree of polarization was expressed in millipolarization units (mP) as calculated by the reader software using fluorescence intensities parallel and perpendicular with the plane of linearly polarized excitation light. 1% DMSO controls were used. Test compounds were assayed in triplicate. Percent inhibition was calculated to express compound activity after normalising to controls.

Alpha Screen Assay

We tested 6x-Histidine-tagged BRCA1 tBRCT, TOPBP1 tBRCT 7/8 or GRB2 SH2, and their cognate biotinylated peptide substrates. Initial checkerboard titrations were carried out to determine the optimal concentration of protein and peptide for each screen. Assays were conducted in Costar 96-well flat bottom white polystyrene plates using assay buffer (20mM Tris buffer pH 7.4, 200mM NaCl, 0.05% Tween-20, 2mM DTT). 5x-working concentrations of the reagents were dispensed in 10μl each in the order: 6x-Histidine tagged protein, biotinylated peptide, test compound. Plates were incubated at room temperature for 20 min and 10 μl each of nickel chelate AlphaLISA® acceptor beads and AlphaScreen® Streptavidin donor beads (both from Perkin Elmer) at a final concentration of 20 μg/ml were added. Plates were covered with adhesive seals and incubated in the dark for 1 h at 25°C. The AlphaScreen® signal was read using TECAN infinite M1000 Pro microplate reader at excitation wavelength 680nm and emission wavelength 520-620nm. 1% DMSO controls were run in parallel, and used to calculate percent inhibition. A 10-point dose response was performed in triplicate to determine IC_{50} values for each compound.

Microscale Thermophoresis (MST)

Protein domains used in the assay were labeled with NT-647-NHS fluorescent dye using the Monolith NT™ Protein Labeling Kit (NanoTemper Technologies). Assays were carried out in 20mM Tris buffer, pH 7.4, with 200mM NaCl, 0.05% Tween-20 and 2mM DTT. For the direct binding assay, 10μl of labeled protein at a final concentration of 20nM was mixed with 10μl of test compound and incubated on ice for 10 min. For the competitive assay, 10μl of labeled protein at a final concentration of 20nM was mixed with 2μM cognate peptide substrate at the EC_{80} concentration determined by prior titration using a 16-point serial dilution by direct-binding MST. For both assays, samples prepared as above were centrifuged at 15000 rpm at 4°C for 10 min and 4μl of the supernatant was loaded into premium glass capillaries (NanoTemper Technologies). MST analysis was performed at MST power of 40% and LED power of 80%, at 22°C temperature using a Monolith NT.115 (NanoTemper Technologies). An initial "Capillary Scan" was performed to scan for fluorescence across the length of the capillary tray to determine the exact position of each capillary before the MST measurement was started. Test compounds were assayed at 16 different concentrations by serial dilution, and data were analysed using NanoTemper analysis software. K_d values were determined using "T-jump + Thermophoresis" settings. The change in thermophoresis between different experimental conditions was expressed as the change in the normalized fluorescence (ΔF_{norm}), which is defined as F_{hot}/F_{cold} (F-values correspond to average fluorescence values between defined areas in the curve under steady-state conditions under control (F_{cold}) or experimental (F_{hot}) conditions. Titration of the non-fluorescent ligand causes a gradual change in thermophoresis, which is plotted as ΔF_{norm} to yield a binding curve, which was then fitted to derive binding constants.

Unimolecular FRET Sensor Assay

HEK293 cells expressing the stably integrated FRET sensor in a tetracycline-inducible system were used. 3.5×10^5 cells/35mm dish were seeded in Matrigel coated plates and expression was induced using doxycycline (1μg/ml) for 48 h. Cells were incubated with compounds for 24 h in serum containing media at 100μM final concentration with 0.5% DMSO. Where indicated, mCherry-BRCA1 tBRCT constructs were transiently transfected in a 4.5:1 ratio of Fugene:DNA and incubated in serum-containing media for 24 h (Fugene HD transfection reagent, Promega). Two h after 16 Gy IR, cells were washed in 1x PBS (pH 7.4) and fixed in 4% PFA at room temperature (RT) for 10'. Cells were mounted with Mowiol (without anti-fade) before imaging. The following excitation and emission wavelengths were used: Ex 402nm / Em 457nm for Tag-BFP; Ex 483nm / Em 506nm for Tag-GFP2 and Ex 594nm / Em 610nm for mCherry. Two methodologies were adapted to calculate FRET efficiencies: Sensitized Emission (SE) and Acceptor Photobleaching (AP). Images acquired as noted below were quantified using HCS studio 2.0 (Thermo Fisher Scientific) to define nuclear morphology and average nuclear intensities.

For sensitized emission FRET measurements, images were acquired using a Zeiss epifluorescence microscope with Apotome using a 40x oil objective. For spectral corrections Tag-BFP and Tag-GFP2 constructs were used independent from the FRET biosensor. Corrections for the extent of spectral cross-talk were calculated and applied as follows (Müller et al., 2013).

Emission crosstalk of constructs encoding the acceptor or donor alone into the FRET channels was calculated as Co-efficient A and Co-efficient B respectively using the following formulae:

Acceptor in FRET channel (Co-efficient A) = Average intensity of Acceptor only using FRET filter set / Average intensity of Acceptor only using acceptor filter set

Donor in FRET channel (Co-efficient B) = Average intensity of Donor only using FRET filter set / Average intensity of Donor only using donor filter set.

Corrections using the two coefficients were then applied to the FRET efficiency equation: FRET efficiency = FRET – (Coefficient A * FRET biosensor using Acceptor filter set] – (Coefficient B * FRET biosensor using Donor filter set].

Data was then represented as % FRET efficiency of mean nuclear intensities from ~300-600 cells per experiment and represented as mean ± SEM.

For acceptor photobleaching FRET measurements, images were independently acquired for donor and acceptor fluorophore channels before acceptor photobleaching using an LSM780 confocal microscope with a 40x oil objective. A region of interest (ROI) was selected within the nucleus and the acceptor was bleached for $\geq$10 cycles to ensure $\geq$60% bleaching efficiency. After effective photobleaching, images were again acquired for both donor and acceptor channels and then FRET efficiencies were calculated (Paster et al., 2009) by measuring mean nuclear intensities obtained using high-content image analysis software HCS studio 2.0 using this formula:

FRET efficiency = (BFP post-bleach – BFP pre-bleach) / BFP post-bleach.

Data were normalized for bleach efficiencies and plotted from ~300-600 cells per experiment and represented as mean ± SEM.

Immunofluorescence Staining for Damage-Induced Foci

HEK293 cells stably harboring plasmids for Tet-inducible expression of tBRCT domains were seeded at 30,000 cells/well on Matrigel-coated 96-well plates, and treated as indicated. Cells were fixed in 2.5% PFA, for 20' at RT and incubated in 1x PBS with 10% FBS plus 0.5% TritonX-100 for 1 h for blocking and permeabilization. Primary antibody staining was performed at the indicated dilutions in PBST-BSA buffer (0.7mg/ml BSA, 0.05% Tween-20 in 1XPBS) for 1h at RT. Cells were then extensively washed in PBST-BSA buffer and stained with goat-anti-mouse Alexa 488 secondary antibody (Invitrogen), at 1:1000 dilution along with 2.5μg/ml DAPI for nuclear staining. CAL-51 clone 60 cells were stained similarly, except that prior fixing, cells were pre-extracted, with CSK buffer [10mM PIPES [pH 6.8], 100mM NaCl, 300mM Sucrose, 3mM $MgCl_2$, 1mM EGTA, 0.5% TritonX100] for 5 minutes in ice and stained with Anti-BRCA1 (Ab-1) (MS110) [OP92, 1:200, Millipore] using procedure as mentioned above. For RPA32 staining, an additional step of pre-extraction with 0.4% NP-40 was done before fixation. Images were acquired on a high-content imaging platform (Cellomics ArrayScan VTI HCS Reader (Thermo Fisher Scientific)) using a 40x objective. On average ~800 fields from 6-well replicates, containing a total of ~10-20K cells were imaged per treatment group and quantified using image analysis software (in-house algorithms using MatLab and commercially available HCS studio 2.0 from Thermo Fisher Scientific). Briefly, nuclear objects were defined, and foci were enumerated for each of the different DDR proteins. Plots were generated to compare control (0 Gy) and 16 Gy irradiated samples for foci number vs. percentage of cells. Cut-off values for foci number per cell specifying the maximal difference between control and irradiated samples were determined to calculate the percentage of cells positive for radiation-induced foci. These cut-off values were used to enumerate changes in the percentage of cells positive for radiation-induced foci with or without inhibitor treatment. Representative images at high magnification showing clusters of foci-bearing cells were taken using a Zeiss LSM780 confocal microscope with a 63x oil objective and 3x optical zooming.

Cell Cycle Profiles

HEK293 cells stably harboring plasmids for Tet-inducible expression of tBRCT domains were seeded on 12-well plates at 1.8 $X10^5$ cells/well. Cells were thymidine blocked and synchronously released into the cell cycle following irradiation at 4Gy, without or with exposure to compounds (at final concentrations with 0.5% DMSO) in serum-containing media for 16 h. Cells were re-suspended in 1x PBS, fixed with 80% ethanol, and permeabilized in buffer containing 0.1% Tween-20 in PBS (PBST), for 20' at RT. DAPI staining was used to quantify nuclear DNA content. Analysis was performed using a Beckman Coulter Gallios analyzer, and quantified using the Dean-Jet algorithm in FlowJo software.

Cell Viability Assays

HEK293 cells stably harboring plasmids for Tet-inducible expression of tBRCT domains were seeded at a density of 60,000 cells/35mm dish. Compound addition or inducible expression of BRCA1 tBRCT were performed as indicated, when cells were ~30% confluent, before exposure to 1Gy irradiation. Cells were replenished with media every 3rd day for 7 days, re-suspended in cell dissociation buffer, which was neutralized in 1x PBS. Cells were mixed thoroughly to ensure single cell suspension before measurement of viability using Calcein AM dye and/or cell counting with a hemocytometer. Cell survival fractions were measured using fluorescence readout of Calcein AM with the Tecan Infinite M1000 Pro reader (485nm excitation and 515nm emission) and data were normalized to untreated controls across treatment groups.

QUANTIFICATION AND STATISTICAL ANALYSIS

All data fitting and statistical analyses were performed using GraphPad version 6.05 for Windows (GraphPad Software Inc, www.graphpad.com). No methods were used to determine whether the data met assumptions of the statistical approach. All the experiments were performed at least with three independent repeats and represented as mean ± Standard deviation. Descriptions of samples, and the exact values of n, are provided in the figure legends. Statistical significance was tested using an unpaired, two tailed t-test. *** $P \leq 0.001$

Cell Chemical Biology, Volume *25*

Supplemental Information

Targeting Phosphopeptide Recognition by the Human BRCA1 Tandem BRCT Domain to Interrupt BRCA1-Dependent Signaling

Jayaprakash Periasamy, Vadiraj Kurdekar, Subbarao Jasti, Mamatha B. Nijaguna, Sanjana Boggaram, Manjunath A. Hurakadli, Dhruv Raina, Lokavya Meenakshi Kurup, Chetan Chintha, Kavyashree Manjunath, Aneesh Goyal, Gayathri Sadasivam, Kavitha Bharatham, Muralidhara Padigaru, Vijay Potluri, and Ashok R. Venkitaraman

SUPPLEMENTAL INFORMATION

Figure S1. Related to Figure 1. Structure-activity relationships of compounds inhibiting phosphopeptide recognition by the BRCA1 tBRCT. The FP assay used for compound library screening was validated by: (A) Testing the ability of an unlabeled BACH1 phosphopeptide (GGSRST(pSer)PTFNK) to compete for binding with an identical but TAMRA-labeled phosphopeptide to BRCA1 tBRCT. (B) Testing the ability of previously reported oligopeptide inhibitors (Yuan et al., 2011) to inhibit the binding of TAMRA-labeled BACH1 phosphopeptide to BRCA1 tBRCT. In both A and B, percent inhibition is plotted against peptide concentration and IC_{50} was calculated. Experiments shown represent the mean of three independent experiments ± SD. (C) Determination of the Z Factor. The dot plot shows FP values for the binding of TAMRA-labeled BACH1 phosphopeptide to BRCA1 tBRCT (9360 wells from 260 assay plates; mean FP bound = 151±18 mP) compared to similar values after the addition of excess unlabelled phosphopeptide (2600 wells from 260 assay plates; mean FP unbound = 41±7mP). Each dot represents the mean from a single plate. The assay Z Factor was calculated to be 0.65 using the equation Z Factor = 1 − (3xSD bound + 3xSD unbound)/(mP bound − mP unbound), where SD is the standard deviation from the mean, and mP is the mean fluorescence polarization. (D) The Alpha screen assay was validated by testing the ability of previously reported oligopeptide inhibitors (Yuan et al., 2011) to inhibit the binding of biotin-labeled BACH1 phosphopeptide to BRCA1 tBRCT. Percent inhibition is plotted against peptide concentration. Experiments shown represent mean of three independent experiments ± SD. (E) Superimposition of the unliganded form (PDB: 1JNX, cyan) and the peptide-bound form (PDB: 3K0K, grey) of BRCA1 tBRCT confirms the absence of conformational changes after substrate engagement. Key residues in the binding pocket are shown as sticks. (F) Two-dimensional representation of the percentage intermolecular interaction between CCBT002 and residues in the BRCA1 tBRCT binding pocket during a 5 ns MD simulation. Hydrogen bond

interactions are shown in magenta, and the hydrophobic and charged pocket residues are shown in green and purple spheres, respectively. The histogram shows the fraction of protein–ligand contacts between CCBT002 and BRCA1 tBRCT through a 5 ns MD simulation with the pocket residues plotted on X-axis, and the interaction fraction plotted on Y-axis. (G) A dose response curve for the interaction between BACH1 phosphopeptide and BRCA1 tBRCT (labeled with NT-647-NHS fluorescent dye) determined by MST. Peptide concentration is plotted on the X-axis against changes in normalized fluorescence (ΔF_{norm}) on the Y-axis. Experiments shown represent mean of three independent experiments ±SD. (H) Dose response curve for the competitive inhibition of the interaction between BACH1 phosphopeptide and BRCA1 tBRCT by the oligopeptide inhibitor, Ac-pSPVF-COOH, measured by MST. Peptide concentration is plotted on the X-axis against changes in normalized fluorescence (ΔF_{norm}) on the Y-axis. Experiments shown represent mean of three independent experiments ±SD.

Figure S1

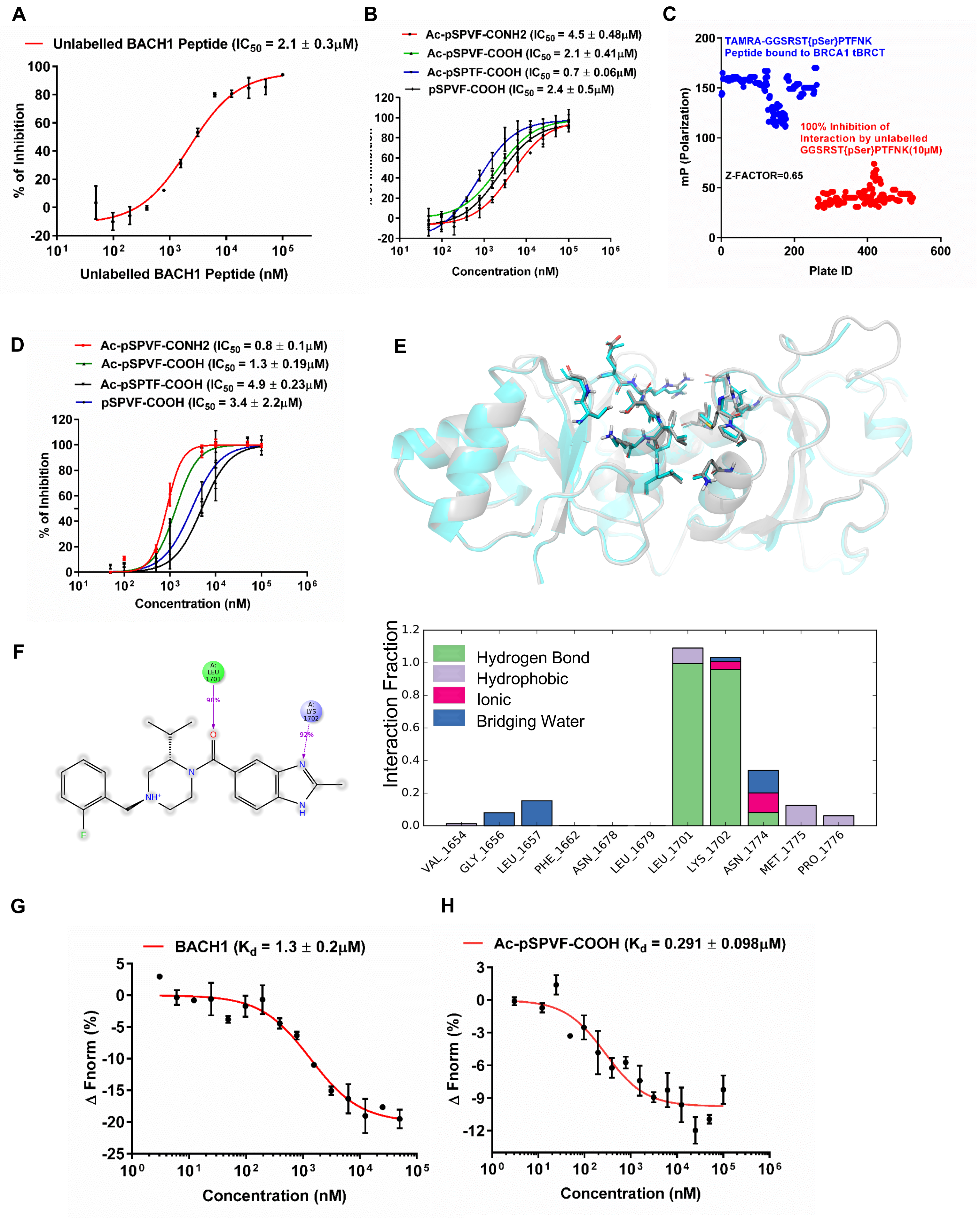
A
Unlabelled BACH1 Peptide (IC50 = 2.1 ± 0.3µM)
% of Inhibition
Unlabelled BACH1 Peptide (nM)
B
Ac-pSPVF-CONH2 (IC50 = 4.5 ± 0.48µM)
Ac-pSPVF-COOH (IC50 = 2.1 ± 0.41µM)
Ac-pSPTF-COOH (IC50 = 0.7 ± 0.06µM)
pSPVF-COOH (IC50 = 2.4 ± 0.5µM)
Concentration (nM)
C
TAMRA-GGSRST{pSer}PTFNK
Peptide bound to BRCA1 tBRCT
mP (Polarization)
100% Inhibition of
Interaction by unlabelled
GGSRST{pSer}PTFNK(10µM)
Z-FACTOR=0.65
Plate ID
D
Ac-pSPVF-CONH2 (IC50 = 0.8 ± 0.1µM)
Ac-pSPVF-COOH (IC50 = 1.3 ± 0.19µM)
Ac-pSPTF-COOH (IC50 = 4.9 ± 0.23µM)
pSPVF-COOH (IC50 = 3.4 ± 2.2µM)
% of Inhibition
Concentration (nM)
E
F
A: LEU 1701
98%
A: LYS 1702
92%
Interaction Fraction
Hydrogen Bond
Hydrophobic
Ionic
Bridging Water
VAL_1654
GLY_1656
LEU_1657
PHE_1662
ASN_1678
LEU_1679
LEU_1701
LYS_1702
ASN_1774
MET_1775
PRO_1776
G
BACH1 (Kd = 1.3 ± 0.2µM)
Δ Fnorm (%)
Concentration (nM)
H
Ac-pSPVF-COOH (Kd = 0.291 ± 0.098µM)
Δ Fnorm (%)
Concentration (nM)

Figure S2, related to Figure 4. Bractoppin inhibits substrate recognition by the BRCA1 tBRCT in the cellular milieu. Effect of Bractoppin (30μM or 100μM) or its inactive analog CCBT2047 (100μM) on FRET measured by sensitized emission. The panel on the left indicates changes in FRET efficiency (mean ± SEM) in cells after 24 h of compound treatment. The second panel on the right, measures FRET efficiency as a dot plot wherein each dot represents a single cell (Control, n= 747; Bractoppin 30μM, 566; Bractoppin 100μM, 789; CCBT2047 100μM, 517). Statistical significance was determined using an unpaired two-tailed t-test *** $p \leq 0.001$.

Figure S2

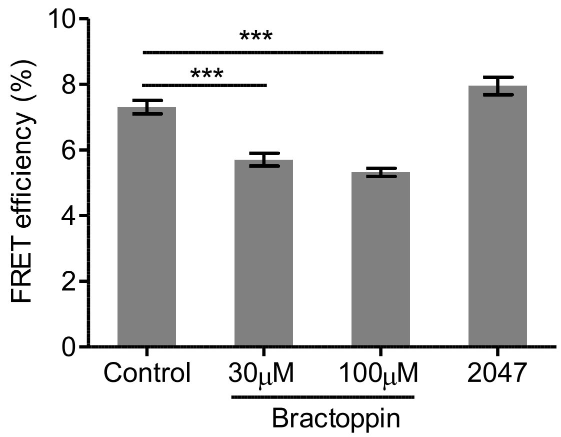

FRET efficiency (%)
10
8
6
4
2
0
***
***
Control
30μM
100μM
2047
Bractoppin

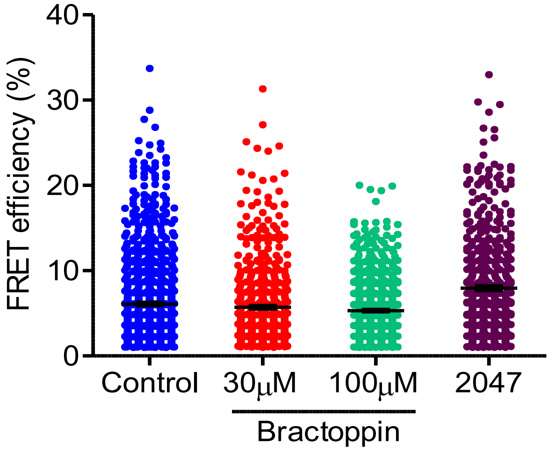

FRET efficiency (%)
40
30
20
10
0
Control
30μM
100μM
2047
Bractoppin

Figure S3, related to Figure 5. Bractoppin selectively inhibits cellular substrate recognition by the tBRCT domain family. Effect of mutations in the BRCA1 tBRCT domains on substrate recognition and the assembly of endogenous BRCA1 foci measured by high-content automated imaging 18 h after the indicated treatments (untreated cells (0 Gy); irradiation alone (16 Gy); Tet-induced expression of wild-type or mutant forms of BRCA1 tBRCT 30h before irradiation). The percentage of cells positive for radiation-induced nuclear BRCA1 foci is shown after expression of (A) wild-type BRCA1 tBRCT (B) mutant BRCA1 tBRCT (M1775R) or (C) mutant BRCA1 tBRCT (S1655A/K1702M). Values are expressed as mean±SD (n= 20000, 0Gy; 15000, 16Gy; 10000, WT BRCA1 tBRCT; 15000, M1775R BRCA1 tBRCT; 15000, S1655A/K1702M BRCA1 tBRCT, Statistical significance *** $P \leq 0.001$). (D) Percentage of CAL-51 clone60 cells positive for radiation-induced nuclear BRCA1 foci (mean±SD; n= 1472, 0Gy; 1789, 16Gy; 2233, Bractoppin;) enumerated by high-content imaging at low-magnification (see Methods). Treatment conditions were as described in Figure 5A. Statistical significance was determined using an unpaired two-tailed t-test. * $p=0.05$ (E) Representative high-content microscopic images depicting recruitment of MDC1 and BRCA1 proteins into nuclear foci in the indicated treatment conditions (untreated cells (0Gy); irradiation alone (16Gy); Tet-induced MDC1-tBRCT expression 24h before irradiation; 100μM Bractoppin or its inactive analog CCBT2047 added 6h after irradiation). Staining was done 18h after irradiation in the 1st to 3rd column (left to right) for DAPI (blue), MDC1 (green), BRCA1 (red) and merged images, respectively. Scale bar, 50μm. (F) Percentage of cells positive for radiation-induced TOPBP1 foci without or after treatment with 100μM Bractoppin or its inactive analog CCBT2047 added 6h after irradiation in U2OS cells. Results were measured and depicted as above. Statistical significance was tested using an unpaired two-tailed t-test. ** $p \leq 0.01$. Similar results were observed in 3 independent repeats.

Figure S3

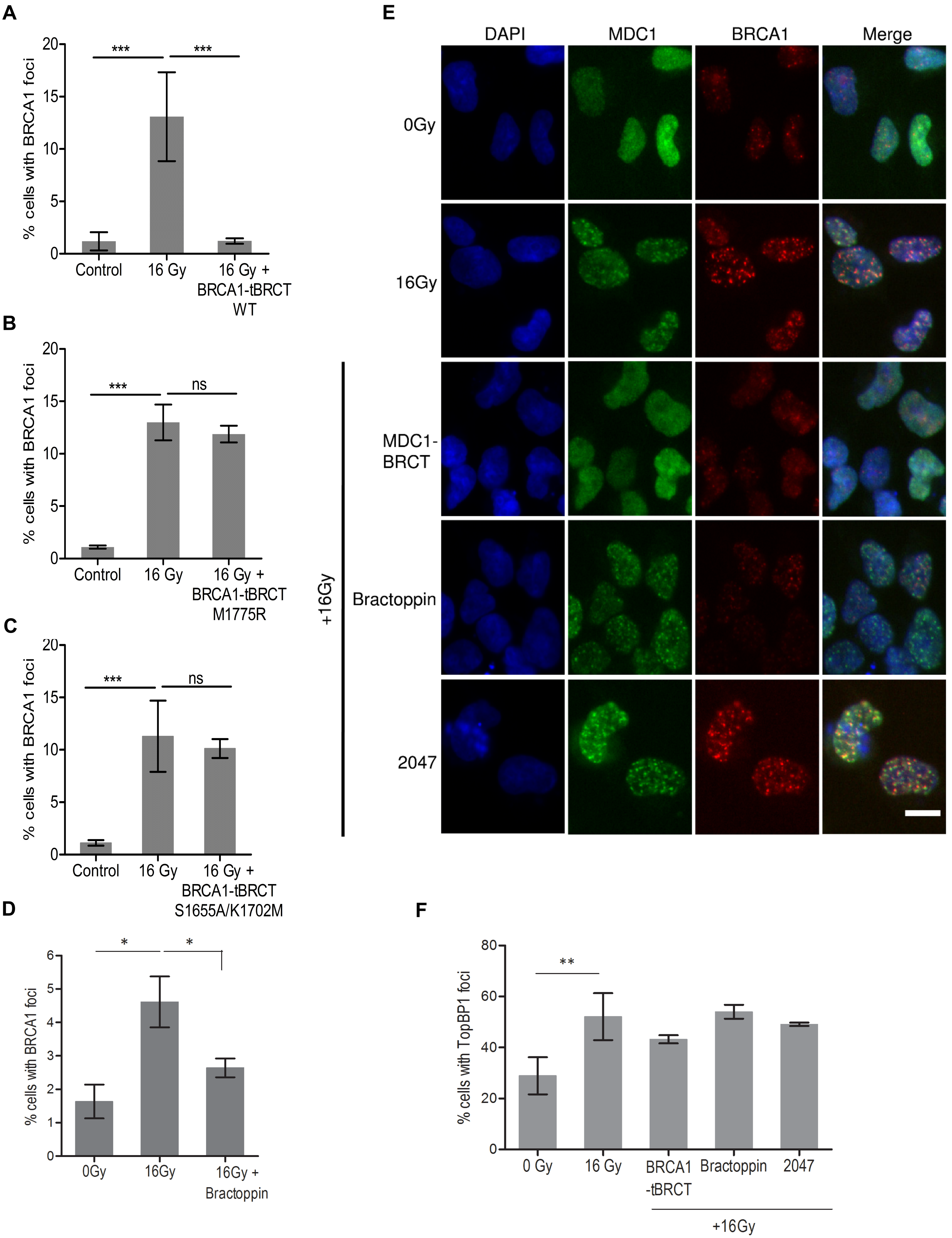
A
% cells with BRCA1 foci
20
15
10
5
0
***
***
Control
16 Gy
16 Gy + BRCA1-tBRCT WT
B
% cells with BRCA1 foci
20
15
10
5
0
***
ns
Control
16 Gy
16 Gy + BRCA1-tBRCT M1775R
C
% cells with BRCA1 foci
20
15
10
5
0
***
ns
Control
16 Gy
16 Gy + BRCA1-tBRCT S1655A/K1702M
D
% cells with BRCA1 foci
6
5
4
3
2
1
0
*
*
0Gy
16Gy
16Gy + Bractoppin
E
DAPI
MDC1
BRCA1
Merge
0Gy
16Gy
MDC1-BRCT
Bractoppin
2047
+16Gy
F
% cells with TopBP1 foci
80
60
40
20
0
**
0 Gy
16 Gy
BRCA1 -tBRCT
Bractoppin
2047
+16Gy

Figure S4. Related to Figure 6. Bractoppin interrupts DNA damage signaling for G2 arrest. Effect of mutations in the BRCA1 tBRCT domains on G2 checkpoint enforcement were measured by flow cytometry after DAPI staining. Cells were irradiated with 4Gy at 8h after synchronous release into the cell cycle from thymidine block, and measurements made 16h later. Wild-type or mutant forms of BRCA1 tBRCT were induced 32h before irradiation. Approximately 15K cells were analyzed per condition, in replicates of 3. (A, B) Percentage of cells (mean ±SD) in the G2/M phase after the indicated treatments. Control, un-irradiated cells; BRCA1 tBRCT S1655A/K1702M double mutant (DM); BRCA1 tBRCT M1775R single mutant (SM). Controls are compared with ± BRCA1 tBRCT DM or SM in the presence of IR. Cells are induced for expression 32h prior to 4Gy IR and measurements are made 16 h post IR. Data quantitatively measures change in accumulation of 4N population (G2+M) across different treatment groups following DAPI staining for its nuclear content. Approximately 15K cells are analyzed per condition, in replicates of 3 using flow cytometer and analyzed using Dean-Jet algorithm for quantitative measurements (mean±SD). Statistical significance was determined using Dunnett's multiple comparisons test post one-way ANOVA. *** P-value: ≤0.001.

(C,D) Cell cycle histograms representing G1 (green), S (yellow) or G2/M (blue) phases after the indicated treatments. Results are representative of 3 independent experiments.

Figure S4

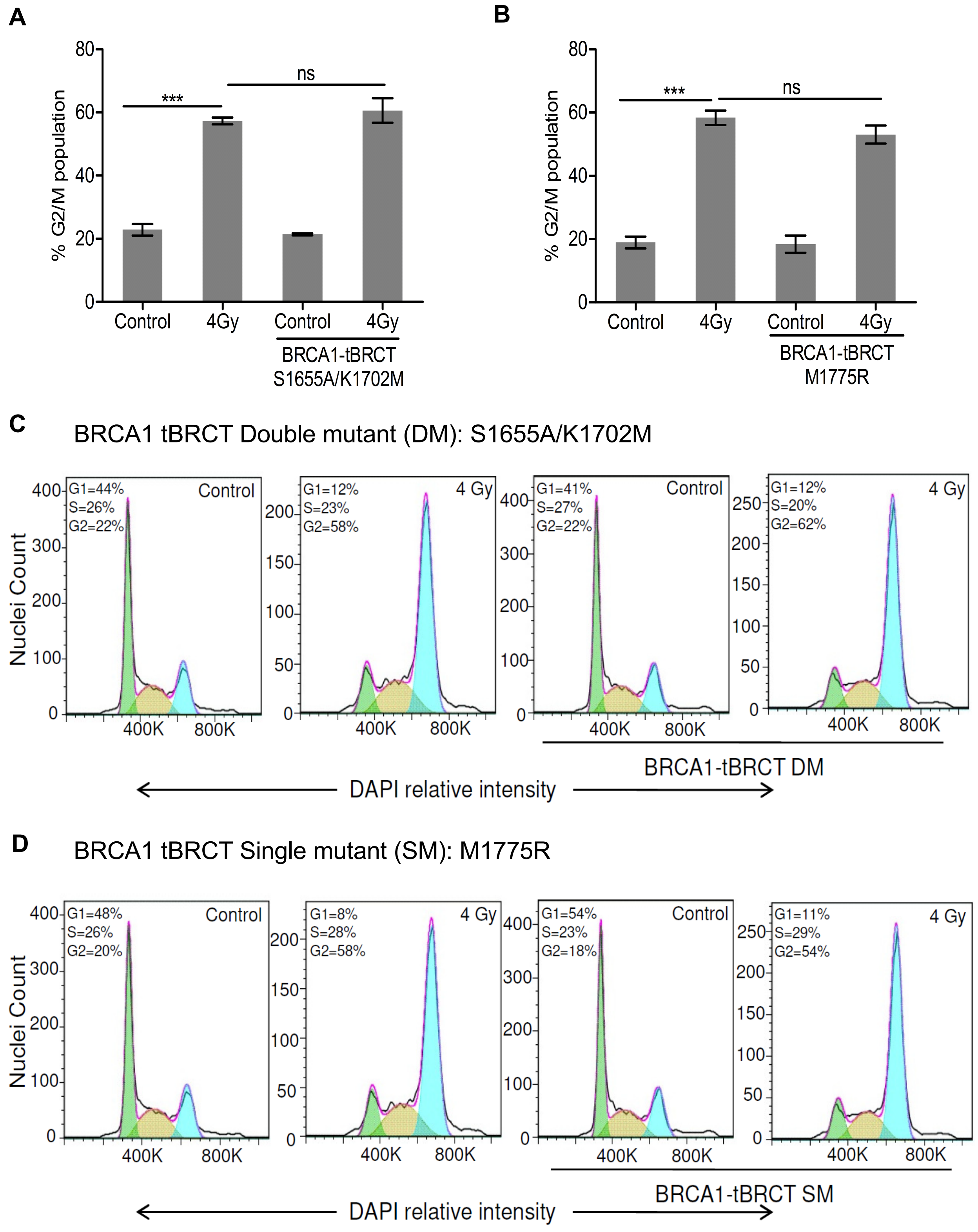

A
% G2/M population
0
20
40
60
80
***
ns
Control
4Gy
Control
4Gy
BRCA1-tBRCT
S1655A/K1702M
B
% G2/M population
0
20
40
60
80
***
ns
Control
4Gy
Control
4Gy
BRCA1-tBRCT
M1775R
C
BRCA1 tBRCT Double mutant (DM): S1655A/K1702M
Nuclei Count
G1=44%
S=26%
G2=22%
Control
G1=12%
S=23%
G2=58%
4 Gy
G1=41%
S=27%
G2=22%
Control
G1=12%
S=20%
G2=62%
4 Gy
400K
800K
BRCA1-tBRCT DM
DAPI relative intensity
D
BRCA1 tBRCT Single mutant (SM): M1775R
Nuclei Count
G1=48%
S=26%
G2=20%
Control
G1=8%
S=28%
G2=58%
4 Gy
G1=54%
S=23%
G2=18%
Control
G1=11%
S=29%
G2=54%
4 Gy
400K
800K
BRCA1-tBRCT SM
DAPI relative intensity
